# Supplementary material for: Clinical Considerations While Providing Care for Patients During Ramadan: A Framework for Health Care Professionals
Source: MedEdPORTAL. 2026 Jun 25;22:11614. doi: 10.15766/mep_2374-8265.11614 (PMC13294187; doi:10.15766/mep_2374-8265.11614)
Supplement: Supplementary file 1 — Facilitator Guide.docxRamadan and the Fasting Patient Module.pptxPreworkshop and Postworkshop Survey.docx [file mep_2374-8265.11614-s001.zip › A. Facilitator Guide.docx]

**Providing Optimal Care for Fasting Patients During Ramadan: A Framework for Healthcare Professionals**

Facilitator Guide

Goal

The primary goal of this module is to increase physician and trainee knowledge of Ramadan and its impact on patient care. The session is designed to increase provider comfort in caring for patients who fast, with emphasis on adapting management plans for those with chronic conditions. By equipping providers with both knowledge and practical frameworks, the module aims to empower them to adjust medical recommendations in alignment with patients’ religious practices and lifestyle changes during Ramadan.

In the long term, this module seeks to strengthen communication between healthcare providers and Muslim patients, fostering trust through culturally responsive care. Supporting patients in observing their religious obligations while managing chronic conditions may improve adherence to treatment recommendations and contribute to better health outcomes during Ramadan.

Required Materials:

- Ramadan and the Fasting Patient Module (Appendix B)
- Computer projector or virtual link

Facilitators were provided with the guide one week prior to the date of the workshop. Additional time for preparation may be required for non-Muslim facilitators who are unfamiliar with workshop content to clear any confusion and prepare for possible questions. Facilitators are encouraged to conduct practice workshops with the guide prior to the actual workshop date to ensure clear communication and steady pacing.

Workshop Objectives:

By the end of this session, participants will be able to...

- Identify the key elements of Ramadan and its religious importance to understand the values of Muslim patients who wish to fast
- Will be able to recognize the clinical implications of fasting for patients with chronic conditions and the need for altered medical advising to control conditions during Ramadan
- Discuss a framework for taking a pertinent history from a patient who wishes to fast
- Generate ideas for altering medical advising to accommodate lifestyle changes during Ramadan

Suggested Agenda and Timetable:

- Slide 1: Introduction – 1 min
- Slide 2: Pre-workshop survey – 3 min
- Slide 3: Objectives – 1 min
- Slide 4: Islam overview – 2 min
- Slides 5-6: Ramadan overview – 4 min
- Slide 7: Fasting exemptions – 2 min
- Slide 8: Typical schedule during Ramadan – 4 min
- Slide 9: Perspectives of individuals with chronic illness – 2 min
- Slide 10: Need for altered advice – 2 min
- Slide 11: Creating knowledgeable physicians – 2 min
- Slides 12-14: Discussion of specific disease affected by fasting – 5 min
- Slide 15: Nutritional advice for fasting patients – 2 min
- Slide 16: Shared decision-making framework – 5 min
- Slides 17-20: Sample cases – 12 min
- Slide 21: Shared decision-making framework – 2 min
- Slide 22: Advising against fasting – 3 min
- Slide 23: Resources – 3 min
- Slide 24: Questions – 2 min (more time may be required depending on engagement)
- Slide 25: Post-workshop Survey – 3 min
- Slide 26: Sources – 1 min

Slide Instructions:

Slide 1: Title and Introduction

“Welcome to ‘Ramadan and the Fasting Patient’. Today we are going to discuss how to accommodate medical advising for patients who want to fast for Ramadan in order to mitigate the risks associated with this practice.”

Facilitators should introduce themselves to the audience including their names, pronouns, and titles/institution.

Slide 2: Pre-workshop survey

Allow participants 3 min to fill out the survey (survey outlined in Appendix C). Help with typing in the link and/or scanning the QR code to access the online survey. Answer any participant questions as they arise.

Slide 3: Objectives

Read the learning objectives with participants:

“Through this workshop, we will…

- Identify the key elements of Ramadan and its religious importance to understand the values of Muslim patients who wish to fast
- Recognize the clinical implications of fasting for patients with chronic conditions and the need for altered medical advising to control conditions during Ramadan
- Discuss a framework for taking a pertinent history from a patient who wishes to fast
- Generate ideas for altering medical advising to accommodate lifestyle changes during Ramadan

Please feel free to ask questions or bring up concerns at any point during the presentation”

Slide 4: Islam Overview

“Islam is the second most common world religion and the fastest growing religion in the world.

In [local county], the percentage of Muslims is [statistic]. Many Muslim patients are also immigrants, which can create an additional language and cultural barrier.”

Add statistics specific to your local community that would make the workshop relevant to your geographic location/community.

Slide 5: Ramadan Overview

“Ramadan is one of the five pillars of Islam. The five pillars include prayer, charity, pilgrimage, belief in God and prophets, and fasting. The dates for Ramadan are based on the lunar calendar; however, we use the solar calendar. As a result, the dates of Ramadan change annually and are about 10 days earlier each year. The dates are based on the moon sightings, so exact date of beginning and end is difficult to predict each year. Precise dates are announced by local Mosques or can be found by a simple online search in the day or two before it begins. Ramadan lasts up to 30 days.

Ramadan is the month in which the Holy Book of Islam, the Qur’an, was revealed. During Ramadan, Muslims attempt to attain a consciousness of God, or *taqwa*, in every moment and action to draw closer to Him.”

Slide 6: Ramadan Overview Continued

“Ramadan is a time for increased prayer, charity, and repentance. It is a time to refrain from all negative character traits. Muslims will spend more time devoted to spirituality, which includes going to the Mosque, which promotes a stronger sense of community.

The part of Ramadan that people are most familiar with it fasting which includes abstaining from food, water, smoking, and sexual contact from dawn to sunset”

Slide 7: Fasting Exemptions

“Fasting is seen as a mandatory practice but there are several exceptions, because to Muslims, God does not want fasting to be a stressful occurrence.

The following groups are excused from fasting altogether: pre-pubescent children, frail elderly people, those with illnesses that would be exacerbated by fasting, and those that lack the capacity to choose to fast.

There are also certain people who are excused but expected to make up those fasts at a later date and this includes those who are traveling, pregnant, nursing, or menstruating.

In the event that fasting poses significant harm, alternatives exist and will be addressed. However, before we do this, we’d like to equip you with the knowledge and tools to support patients should they be motivated and passionate about observing Ramadan. The goal is to partner with patients in identifying safe, individualized strategies to enable fasting whenever possible.”

Slide 8: Typical Schedule During Ramadan

“While not every Muslim practices the same way and may participate in all of these, these are the standard times for eating and prayer during Ramadan that could affect the day-to-day lifestyle of observant Muslims patients during Ramadan.

The first meal is the pre-dawn meal. This requires people to wake up prior to sunrise to eat. This meal is called Suhoor, or sehri, depending on the language and/or region people are from.

Moving forward, the events in red text represent times during a fast, when someone would not be eating.”

Advance slide to show next icon

“The first prayer of the day is called Fajr. This is the time when the fast begins, so suhoor needs to be finished before then.”

Advance slide to show next icon

“Dhuhr is the second prayer and occurs in the middle of the day. Asr is the third prayer that occurs at mid-afternoon.”

Advance slide to show next icon

“At sundown is the fourth prayer, Maghrib, which is also time to break fast. The time to break fast is called Iftar (versus the prayer at the same time, which is Maghrib).”

Advance slide to show next icon

“The last prayer is after sundown and is called Isha. During Ramadan, people often do additional longer prayers at the Mosque called Taraweeh, keeping Muslims up later at night. During the pandemic, some Mosques started offering Taraweeh at home where you can stream the service and allows people to do their prayers at home.

There are diverse ways that Muslims may practice Islam, similar to other Abrahamic religions. For example, some may fast during Ramadan but not complete the five daily prayers or vice versa. It is important to ask open ended questions and avoid judgement when discussing religious practice with patients.”

Slide 9: Perspectives of Individuals with Chronic Illness

“Several studies have shown that there is a trend amongst Muslim patients with chronic disease that is they feel they are low risk and feel that their physician is uneducated about the responsibility of fasting, they will choose to fast against advisement of their physician. Focused interviews conducted with adult patients with chronic illnesses in Texas yielded these viewpoints, which seem to summarize the general feelings expressed throughout the interviews.

A 53-year-old woman said ‘Our diabetes is not in an advanced stage; we can still fast, but if you are in an advanced stage or if you have pain, you cannot fast’, showing that she understands that if it was bad enough, she would not fast, but she would go against the advisement of her physician if she feels up to it.”

Advance slide to show next graphic

“A 69-year-old man said “The doctor tells you to eat… that fasting is dangerous for your kidneys and eyes. But I say that I am my own doctor. I will try it; if I can… and if not, then it is the will of Allah’ showing that he feels this is an obligation for him and he will fast as long as currently feels up to it”

Slide 10: Need for Altered Advice

“Patients who decide to fast for Ramadan against medical advice will often take matters in their own hands or consult people other than medical professionals for advice on managing their conditions during Ramadan. This leads them to seeking guidance from family, friends, or religious leaders over healthcare professionals.”

Advance slide to show next graphic

“Patients will change their own medication regimens or stop taking them in order to fit their schedules during Ramadan.”

Advance slide to show next graphic

“Retrospective reviews of billing statements and EMRs in Massachusetts found that during Ramadan, Muslims had higher numbers of hospitalizations, primary care visits, and emergency room visits than non-Muslims.

You can compare this to a study measuring adherence to medication for latent TB in Tarrant County, Texas where they use a home delivery system to provide medication. They found that during Ramadan, Muslim patients had much lower completion rates of their antibiotics compared to non-Muslims. However, when they instituted a program to deliver the medications after dusk, they found that completion rates of antibiotics were comparable between Muslims and non-Muslims during Ramadan. This shows that Muslims patients can be adherent to treatment and have improved outcomes if advice is tailored to help them fulfill religious obligations.”

Slide 11: Creating Knowledgeable Physicians

“Most physicians are currently unfamiliar with fasting practices and tend to not accommodate medical advising for Ramadan.

It's really important to be knowledgeable about diverse patient experiences because it allows us to tailor out advice to fit their lifestyles and have better adherence. Literature has reported that patients are more likely to take the advice of healthcare professionals when they show they are informed about their practices and more likely to open up to their providers about what they plan to do.”

Slide 12: Addressing Diabetes for Patients Fasting During Ramadan

“We are going to transition into discussing how some specific conditions are affected by fasting.

The first is diabetes. There are a number of physiologic changes that occur during fasting including metabolic changes, circadian/endocrine changes, insulin sensitivity, and glucose variability due to altered eating and sleeping schedule.

We can advise patients differently by encouraging them to check their blood sugars more regularly/at different timings that align more with their eating schedule, creating a nutrition plan to ensure that patients get all of the nutrients and energy they need to sustain a fast, and possible medication changes that better suit the lifestyle that a patient chooses to lead during Ramadan.”

Slide 13: Addressing Chronic Kidney Disease (CKD) for Patients Fasting During Ramadan

“Next, we are discussing chronic kidney disease. Fasting affects changes in ion intake which will affect kidney function as well as leads to lifestyle changes that can also affect the secretion of hormones that affect GFR and blood pressure. As a result, we can see patients with electrolyte imbalance, hyperosmolarity, and circadian and endocrine changes.

Changes that we might want to make for patient plans are changing diuretics since patients won’t be drinking water throughout the day, encouraging them to drink a specific amount of water when they can and setting goals for how much water to drink throughout the night, and nutritional advising to ensure electrolyte balance.”

Slide 14: Addressing Pregnancy for Patients Fasting During Ramadan

“Lastly, we are discussing pregnancy. While I said at the beginning that pregnant people may be excused from pregnancy, nearly 99% of pregnant women in Bangladesh fast, so it is likely that there are many pregnant women who will want to fast and we need to ensure that we offer proper guidance and monitoring.

Physiologic changes that might be seen include metabolic changes, circadian and endocrine changes, and decreased placental and uterine blood flow.

For advising, we can, again, offer nutritional guidance to ensure that mother and baby get the nutrients and calories they both need to sustain growth, critically analyze the risk associated with fasting in each individual pregnancy based on complications, and monitor more intensely than we might do at a particular gestational age.”

Slide 15: Nutrition Advice for Patients Fasting for Ramadan

“Here is some general nutritional advice for all patients who wish to fast in order to keep their energy up during the day and prevent crashes. First, patients should strive to eat a well balanced suhoor and iftar. This includes eating plentiful fruits, vegetables, protein sources, and unsaturated fats. This will ensure they are able to get all of their nutrients when they can eat and not miss any food groups while eating fewer meals. Patients should prioritize eating complex carbohydrates like barley, oats, wheat, beans, and lentils over simple carbohydrates. This will help them have more sustained energy during the day, rather than energy bursts. This can be exchanged for typical rice or breads, or they can find traditional recipes that already incorporate these ingredients. Along the same lines, they should be encouraged to eat foods that are high in fiber such as bran, whole grains, and fruits. Fiber slows digestion which also helps provide sustained energy throughout the fast. They should try to cook with monosaturated oils, like canola or olive oil, as these increase HDL and lower LDL as well as aid in the absorption of vitamins A, D, E, and K. You should work with your patient to set a goal of how much water to drink per day to ensure that they are aggressively hydrating and preventing dehydration and hypovolemia. Lastly, they should avoid caffeine, fatty foods, processed foods, and food with high glycemic indices because these will provide glucose spikes, and subsequent crashes.”

Slide 16: Shared Decision-Making Framework

“We are going to discuss a framework for taking a history from a patient who wishes to fast that will help assess the risk associated with fasting to open the doors for a conversation between you and the patient on if fasting is a feasible option for them and accommodations that can be made to help them sustain a fast.

The first thing to review is past medical history and morbidities to look into the severity and complexity of existing disease.”

Advance slide to show next icon

“You should discuss anticipated lifestyle changes with the patient. Consult with the patient what their day typically looks like while fasting including meal times, sleep habits, workout routines, keeping in mind that not all Muslims practice the same ways.”

Advance slide to show next icon

“You should discuss the patient’s values. Discuss how important fasting is to them personally, their preferences, concerns, beliefs, etc.”

Advance slide to show next icon

“You should ask about the location they plan to be in during Ramadan. Sunrise and sunset times change based on latitude. Additionally, areas with different weather patterns may also make it more difficult to fast, for example expelling more energy to keep the body warm in winter or cool in summer.”

Advance slide to show next icon

“You should inquire about previous fasting experiences. Patients can try 3-5 days of trial fasts a month before Ramadan to see how they fare and discuss adjustments to make based on experiences.”

Advance slide to show next icon

“You should review medications, timings, and administration routes to see if anything needs to be changed based on changed lifestyle. Seek input from specialists when necessary to ensure safe medication changes.”

Advance slide to show next icon

“You should talk to patients about red flags while fasting and when they would consider breaking their fast and seeking medical help because it has become dangerous to continue.”

Advance slide to show next icon

“Lastly, you should consult resources from expert organizations such as ACOG, ACP, AAFP, etc.”

Slide 17: Large Group Sample Case

Review the case as a large group.

“We have a few sample cases. The information has been laid out following the framework we just discussed.

‘A 60-year-old man with a history of hypertension and stage 2 chronic kidney disease (CKD) is seen in the clinic for a routine follow-up. His blood pressure is well-controlled, and his recent laboratory results show a stable estimated glomerular filtration rate (eGFR). The patient expresses his desire to fast during Ramadan. He is concerned about the effects of fasting on his kidney function and blood pressure management.’”

Advance slide to show more text

“For lifestyle changes, he plans on working the same hours but will likely be getting less sleep in order to pray Taraweeh and eat Suhoor.”

Advance slide to show more text

“This patient expresses a desire to fast as long as it is safe to do so.”

Advance slide to show more text

“He views this as a religious obligation he must fulfill.”

Advance slide to show more text

“He plans to be in Albany, NY where Fajr is around 5:30 am and Maghrib is around 7:15 pm right now.” - This can be altered to reflect location and current sunrise/sunset times

Advance slide to show more text

“He currently takes ramipril and amlodipine. As a group, discuss what simple suggestions you would advise for this patient to change disease management while fasting for Ramadan.”

Give participants about 2 minutes to brainstorm and discuss ideas. Have them share ideas with the group and discuss the benefits and drawbacks of each suggestion together.

Slide 18: Large Group Sample Case Continued

“Thank you for your great suggestions.”

Advance slide to show more text

“The simple answer that we had listed was encouraging the patient to drink 2.5-3 L of fluids during non-fasting hours. Consider optimizing hydration with electrolyte rich sources such as coconut water or sports drinks. Advise that extra care should be taken if fasts are long or in hot weather. This is one suggestion but there are many good ways to manage this patient that does not create excessive strain or burden.”

Slide 19: Small Group Case #1

Review the case together but participants will discuss the case in small groups of 3-4 people before sharing altogether. Groups can be formed based on the people in close proximity to each other in the audience, and audience members may self-assemble into groups.

“’A 45-year-old woman with a history of hypothyroidism is seen in the clinic for a follow-up appointment. She was recently started on levothyroxine therapy. The patient plans to fast during Ramadan, which involves abstaining from food and drink from dawn until sunset. She is unsure how to take her medication during this time. She is currently taking her medication first thing in the morning on an empty stomach.’ What recommendation would you make for this patient? Discuss in groups of 3-5 people”

Give participants 3 minutes to brainstorm and discuss ideas. After 3 minutes, have groups share their suggestions altogether.

Advance slide to show more text

“The recommendation we had listed was to set an alarm for 1 hr to 30 min before waking up for Suhoor and Fajr to take medication as this does not put excessive strain on the patient as they can go back to sleep after taking the pill and still allows for absorption of their medication.”

Slide 20: Small Group Case #2

Review the case together but participants will discuss the case in small groups before sharing altogether

“’A 55-year-old man with type 2 diabetes is being seen for a routine follow-up. His current medications include glyburide and metformin. He plans to fast during Ramadan, which involves refraining from food and drink from dawn until sunset. He is concerned about maintaining his blood glucose levels and avoiding hypoglycemia during the fast. His recent HbA1c is 7.5%, and he has a history of mild hypoglycemia when meals are delayed.

What changes can be made to his diabetes management to reduce the risk of hypoglycemia during fasting?’”

Give participants 3 minutes to brainstorm and discuss ideas. After 3 minutes, groups share their suggestions altogether.

Advance slide to show more text

“The recommendation we had listed was to switch to thiazolidinedione or DPP-4 inhibitors, which rarely causes hypoglycemia, 4-8 weeks before fasting. This is one suggestion but, again, there are many ways to manage this patient that does not create excessive strain or burden such as nutritional guidance to increase complex carbohydrates and proteins, avoiding intense exercise, and regular blood sugar monitoring throughout the day (a good way to help patients remember is to encourage them to check with each daily prayer).”

Slide 21: Shared Decision-Making Framework

“This is a summary that represents the things we have discussed so far. You can see that discussing history, lifestyle, values, previous experiences, medications, and location all factor into determining a patient’s risk. After risk is considered, you should discuss safety, support system, accommodations, and alternatives to fasting with your patient based on your judgement of what they can handle.”

Slide 22: Advising Against Fasting

“Like we discussed, you should evaluate the risk associated with fasting. Examples of high risk conditions include things like stage 4-5 CKD, well controlled T1D, or advanced heart failure/MI in the last six months. You can have the patient document trial fast to aid in your evaluation, where they try to fast for about 3 days in the preceding month before Ramadan and see how it goes. If all is well, more can be tried later on.

If you deem it is unsafe for the patient to fast, approach the situation with empathy and explain that, even knowing how important this is, you still do not recommend the patient fast because it will be dangerous.

Lastly, you can recommend they explore alternatives to fasting. This can include make up fasts, donations to charity, or extra prayers. Because you are not the authority on this subject, encourage them to discuss with religious figures to find a solution within their means.”

Slide 23: Resources for Advice/Guidelines

“Here we linked resources from expert organizations on managing conditions during Ramadan. The first is from AAFP on general chronic disease management. The next is from AJOG on managing pregnancy while fasting. The next is from the British Islamic Medical Organization that has information on a variety of conditions. The last is from Diabetes Research and Clinical Practice with information specific to diabetes management.”

Allow participants 3-5 minutes to scan QR codes for linked resources. Help with technical issues when possible. Participants will be able to read these resources independently to address patient questions they might come across in their practice.

Slide 24: Questions

Provide an opportunity for open questions and answers.

Slide 25: Post-workshop survey

Allow participants 3 min to fill out the survey (survey outlined in Appendix C). Help with typing in the link and/or scanning the QR code to access the online survey. Answer any participant questions as they arise.

Slide 26: Sources

“These are the references for the development of this presentation. We encourage you to continue learning and do your own research as well.”
