# Supplementary material for: Clinical Considerations While Providing Care for Patients During Ramadan: A Framework for Health Care Professionals
Source: MedEdPORTAL. 2026 Jun 25;22:11614. doi: 10.15766/mep_2374-8265.11614 (PMC13294187; doi:10.15766/mep_2374-8265.11614)

## Slide 1
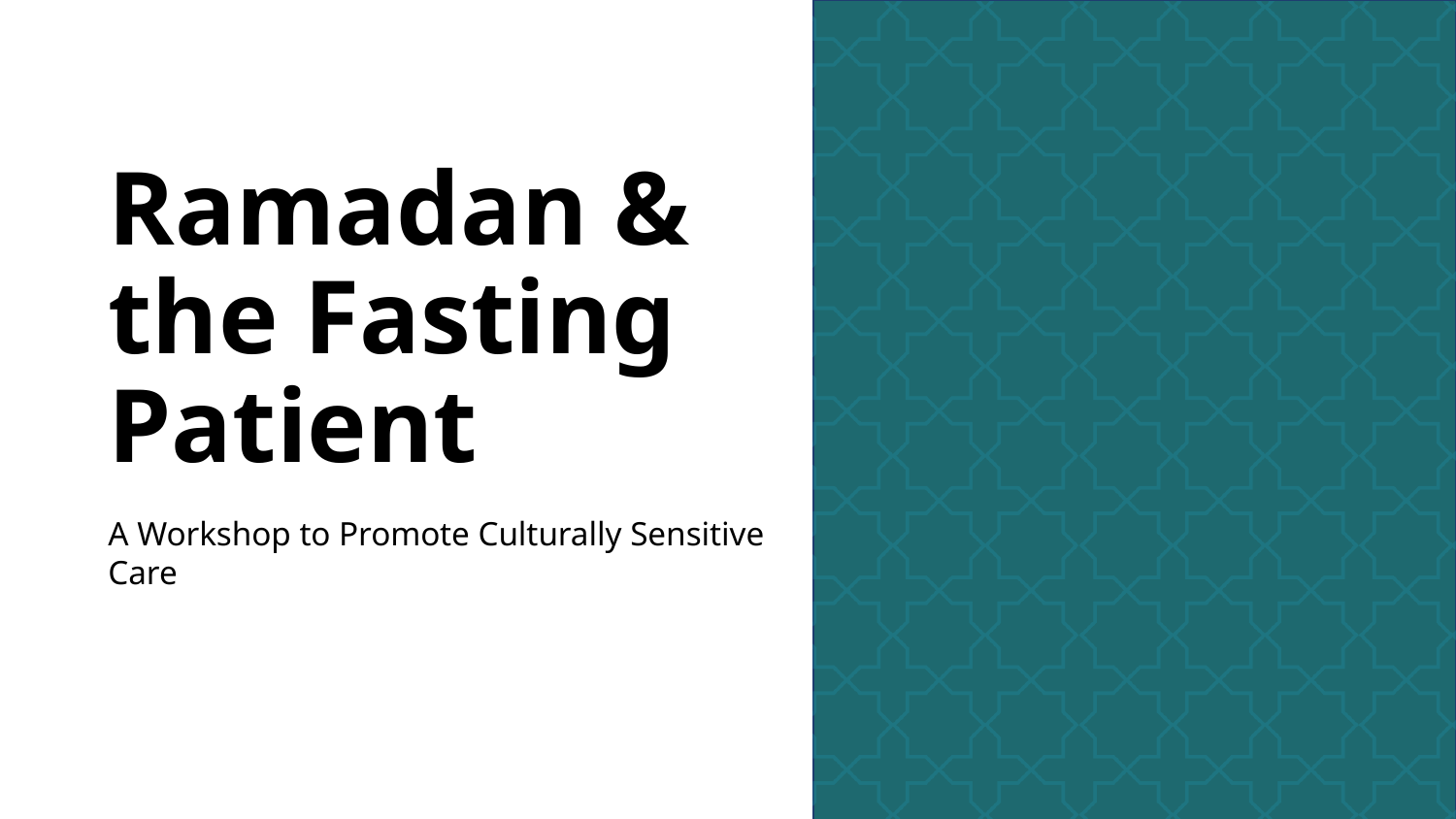

# Ramadan & the Fasting Patient
A Workshop to Promote Culturally Sensitive Care

## Slide 2
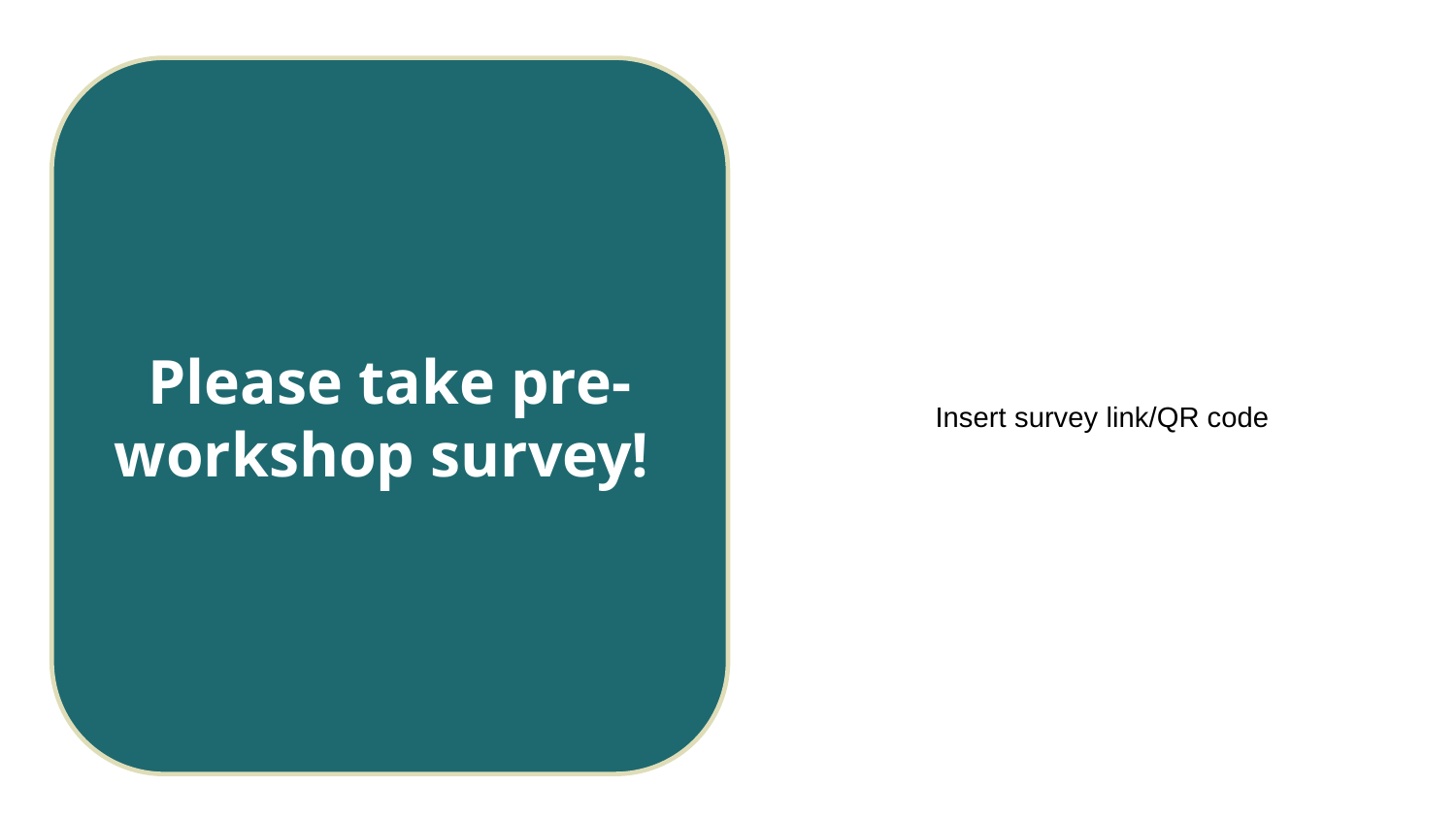

Please take pre-workshop survey!
Insert survey link/QR code

## Slide 3
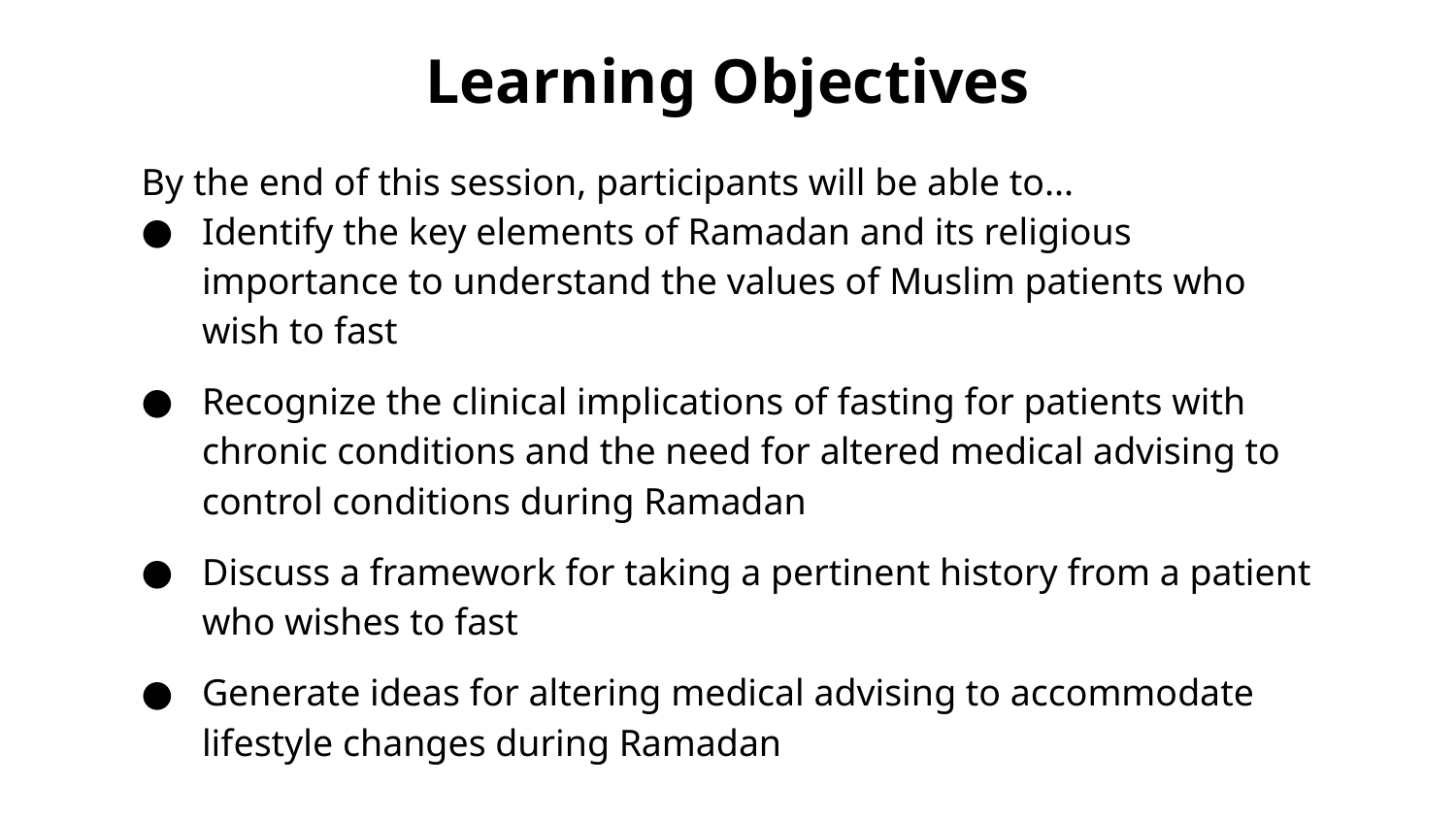

# Learning Objectives
By the end of this session, participants will be able to...
Identify the key elements of Ramadan and its religious importance to understand the values of Muslim patients who wish to fast
Recognize the clinical implications of fasting for patients with chronic conditions and the need for altered medical advising to control conditions during Ramadan
Discuss a framework for taking a pertinent history from a patient who wishes to fast
Generate ideas for altering medical advising to accommodate lifestyle changes during Ramadan

## Slide 4
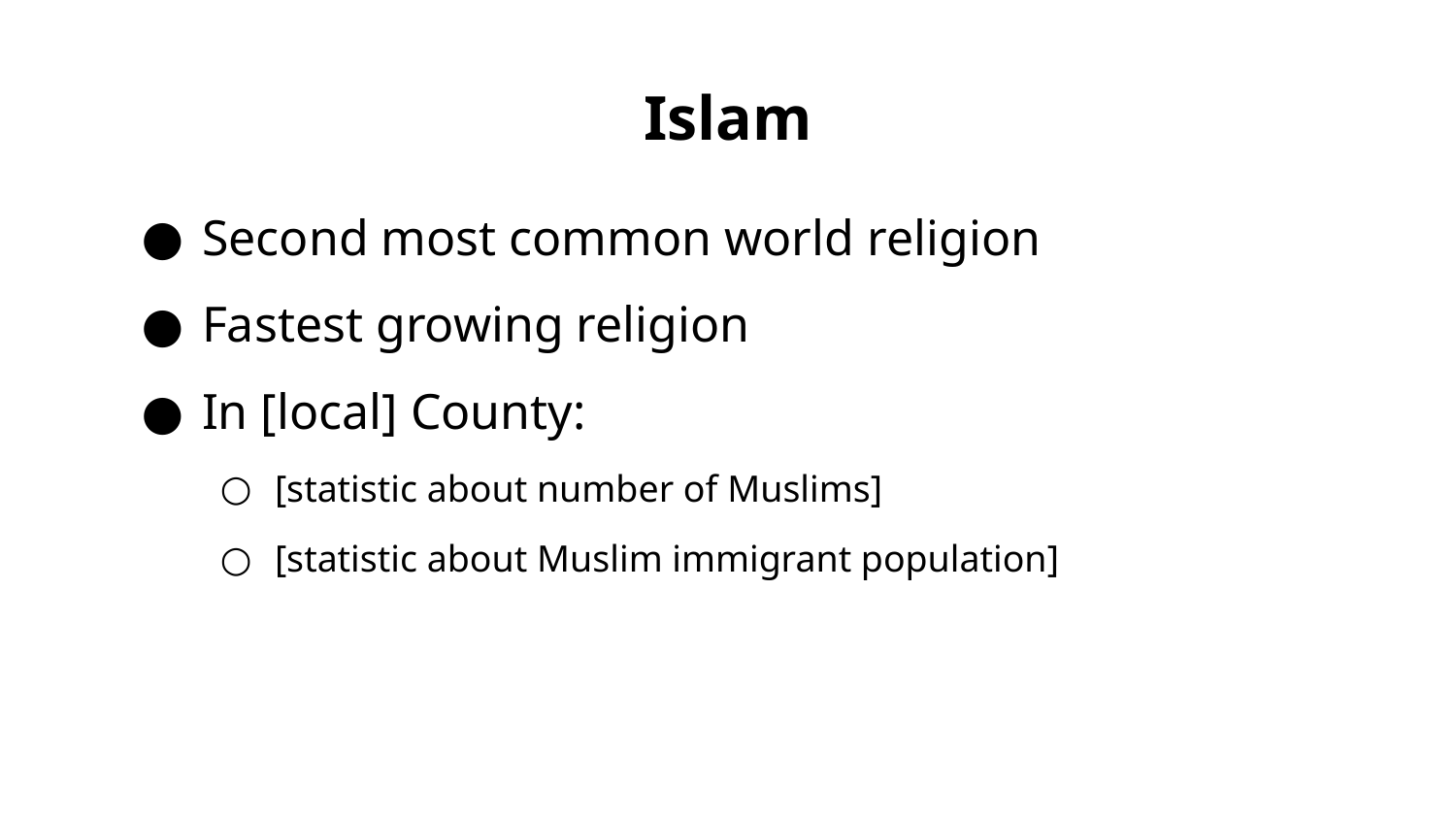

# Islam
Second most common world religion
Fastest growing religion
In [local] County:
[statistic about number of Muslims]
[statistic about Muslim immigrant population]

## Slide 5
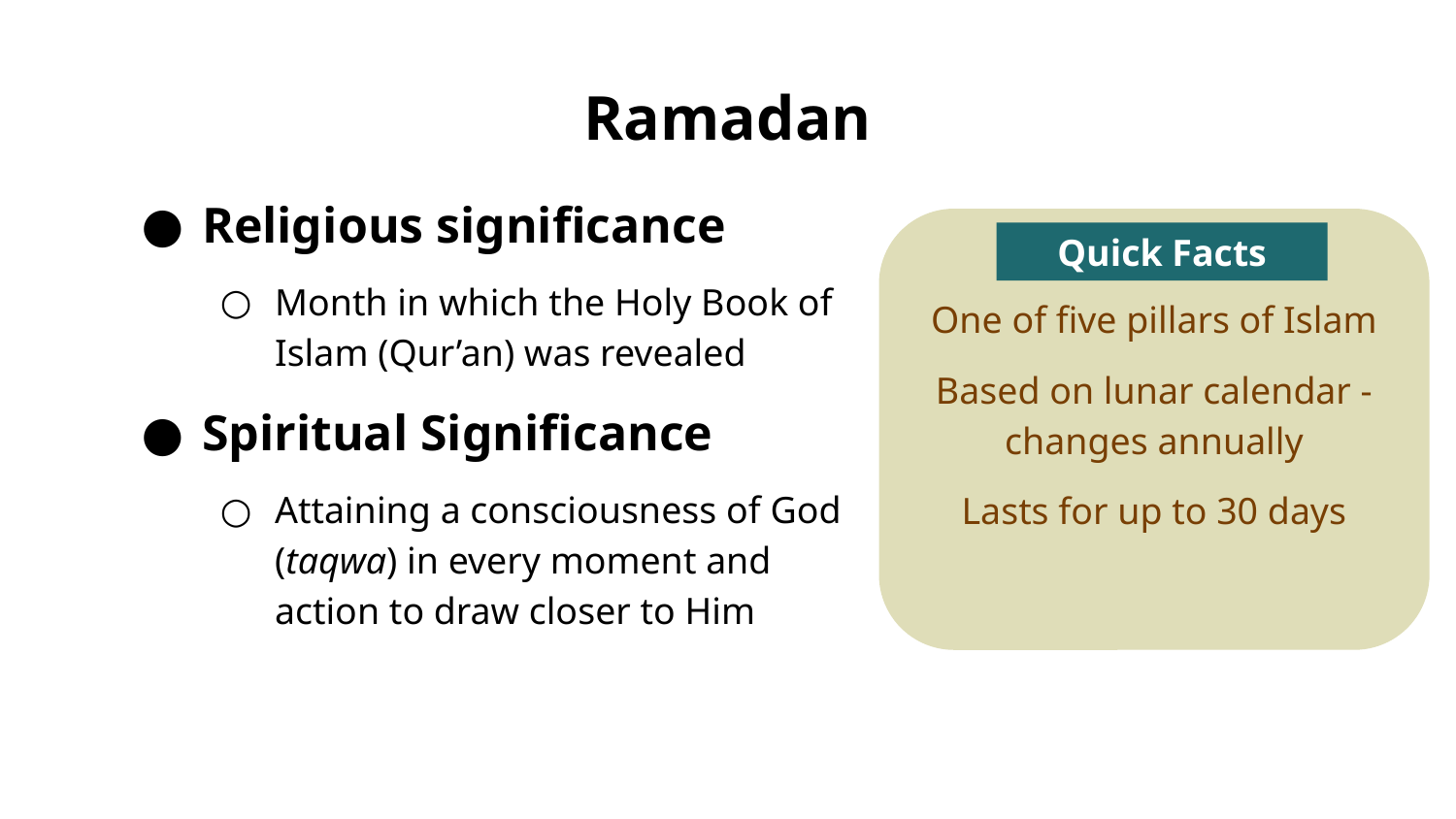

# Ramadan
Religious significance
Month in which the Holy Book of Islam (Qur’an) was revealed
Spiritual Significance
Attaining a consciousness of God (taqwa) in every moment and action to draw closer to Him
Quick Facts
One of five pillars of Islam
Based on lunar calendar - changes annually
Lasts for up to 30 days

## Slide 6
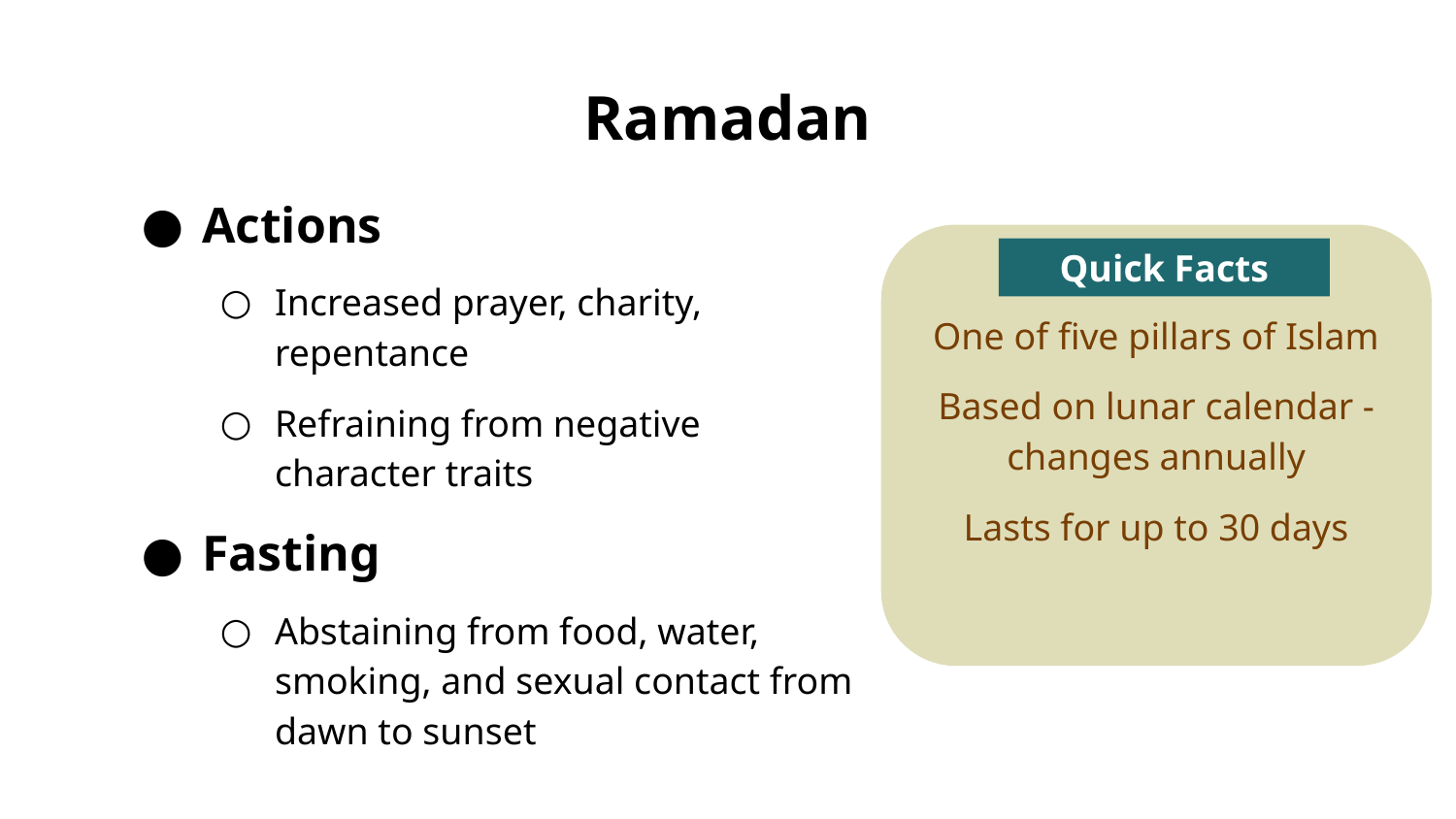

# Ramadan
Actions
Increased prayer, charity, repentance
Refraining from negative character traits
Fasting
Abstaining from food, water, smoking, and sexual contact from dawn to sunset
Quick Facts
One of five pillars of Islam
Based on lunar calendar - changes annually
Lasts for up to 30 days

## Slide 7
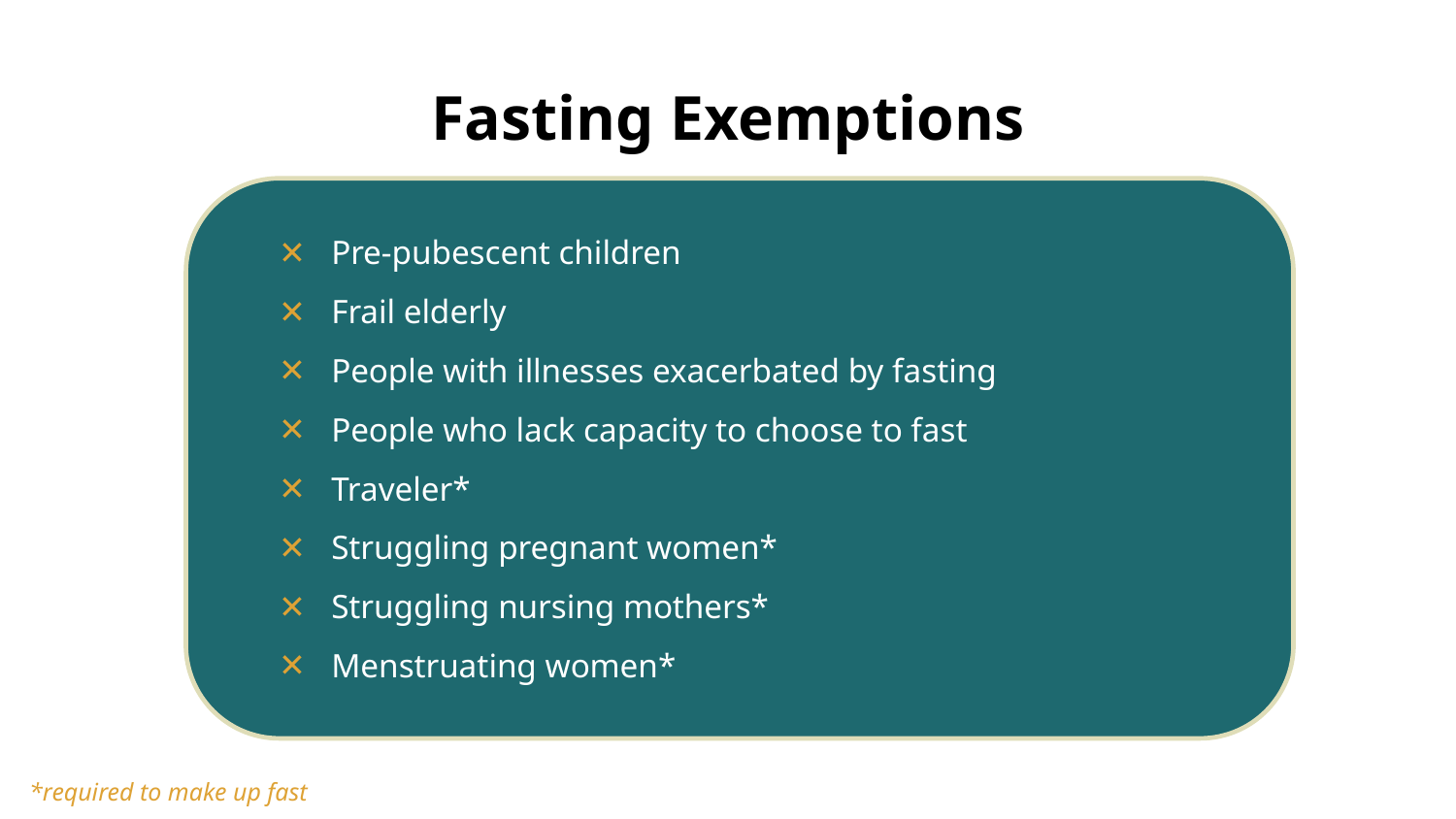

# Fasting Exemptions
Pre-pubescent children
Frail elderly
People with illnesses exacerbated by fasting
People who lack capacity to choose to fast
Traveler*
Struggling pregnant women*
Struggling nursing mothers*
Menstruating women*
*required to make up fast

## Slide 8
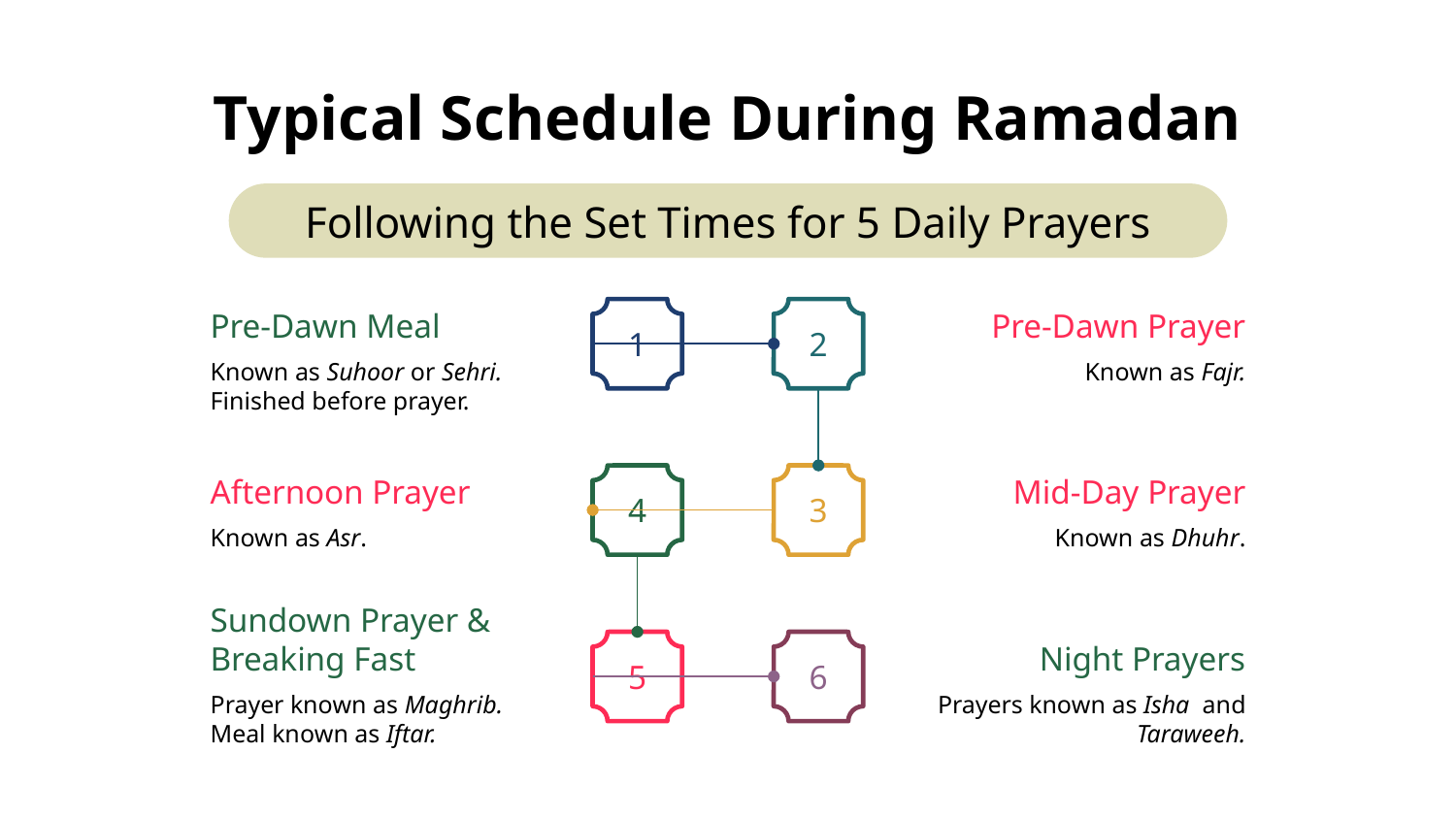

# Typical Schedule During Ramadan
Following the Set Times for 5 Daily Prayers
Pre-Dawn Meal
1
2
Pre-Dawn Prayer
Known as Suhoor or Sehri. Finished before prayer.
Known as Fajr.
4
3
Mid-Day Prayer
Afternoon Prayer
Known as Dhuhr.
Known as Asr.
5
6
Night Prayers
Sundown Prayer & Breaking Fast
Prayers known as Isha and Taraweeh.
Prayer known as Maghrib. Meal known as Iftar.

## Slide 9
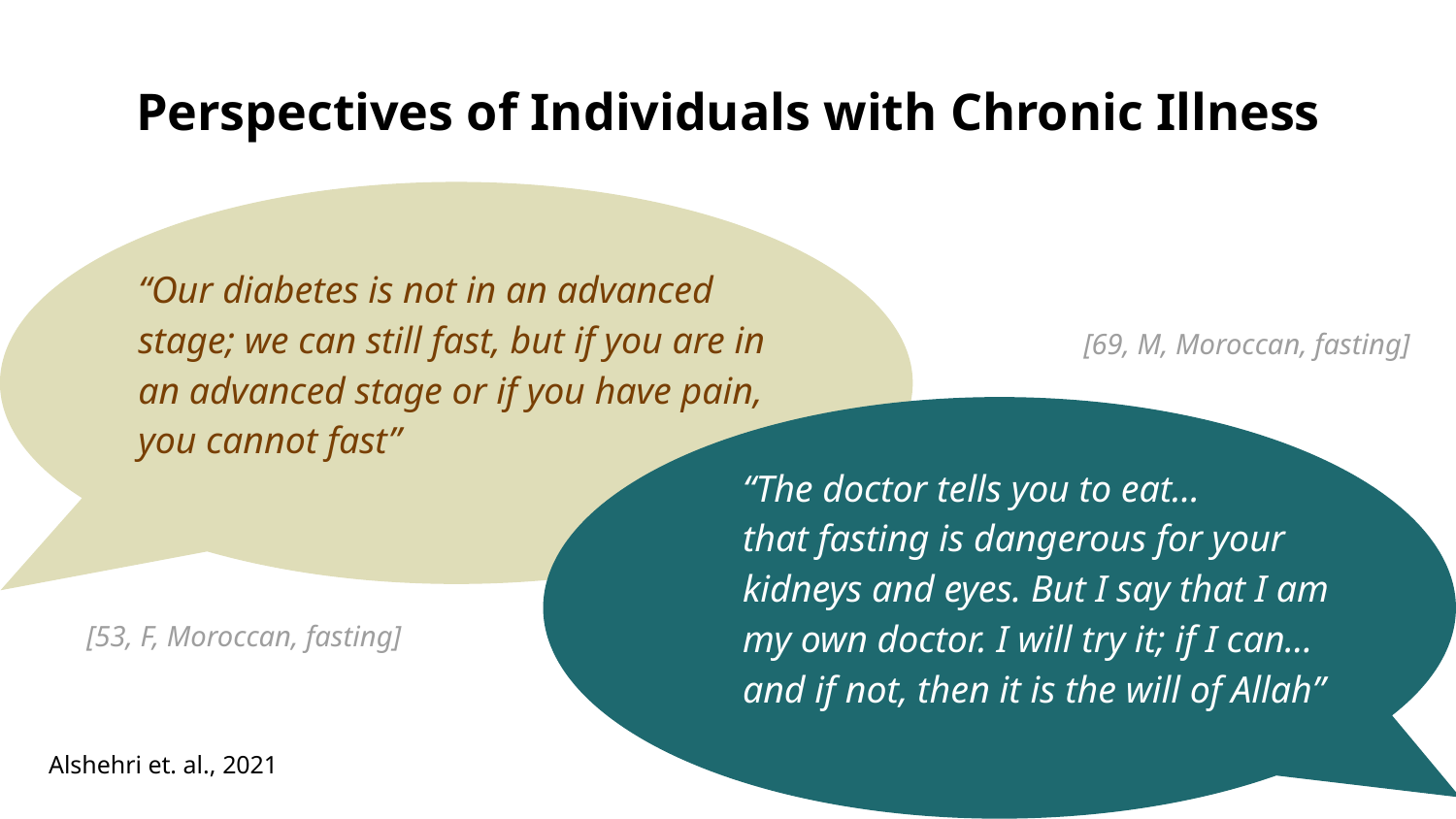

# Perspectives of Individuals with Chronic Illness
“Our diabetes is not in an advanced stage; we can still fast, but if you are in an advanced stage or if you have pain, you cannot fast”
[69, M, Moroccan, fasting]
“The doctor tells you to eat…
that fasting is dangerous for your kidneys and eyes. But I say that I am my own doctor. I will try it; if I can… and if not, then it is the will of Allah”
[53, F, Moroccan, fasting]
Alshehri et. al., 2021

## Slide 10
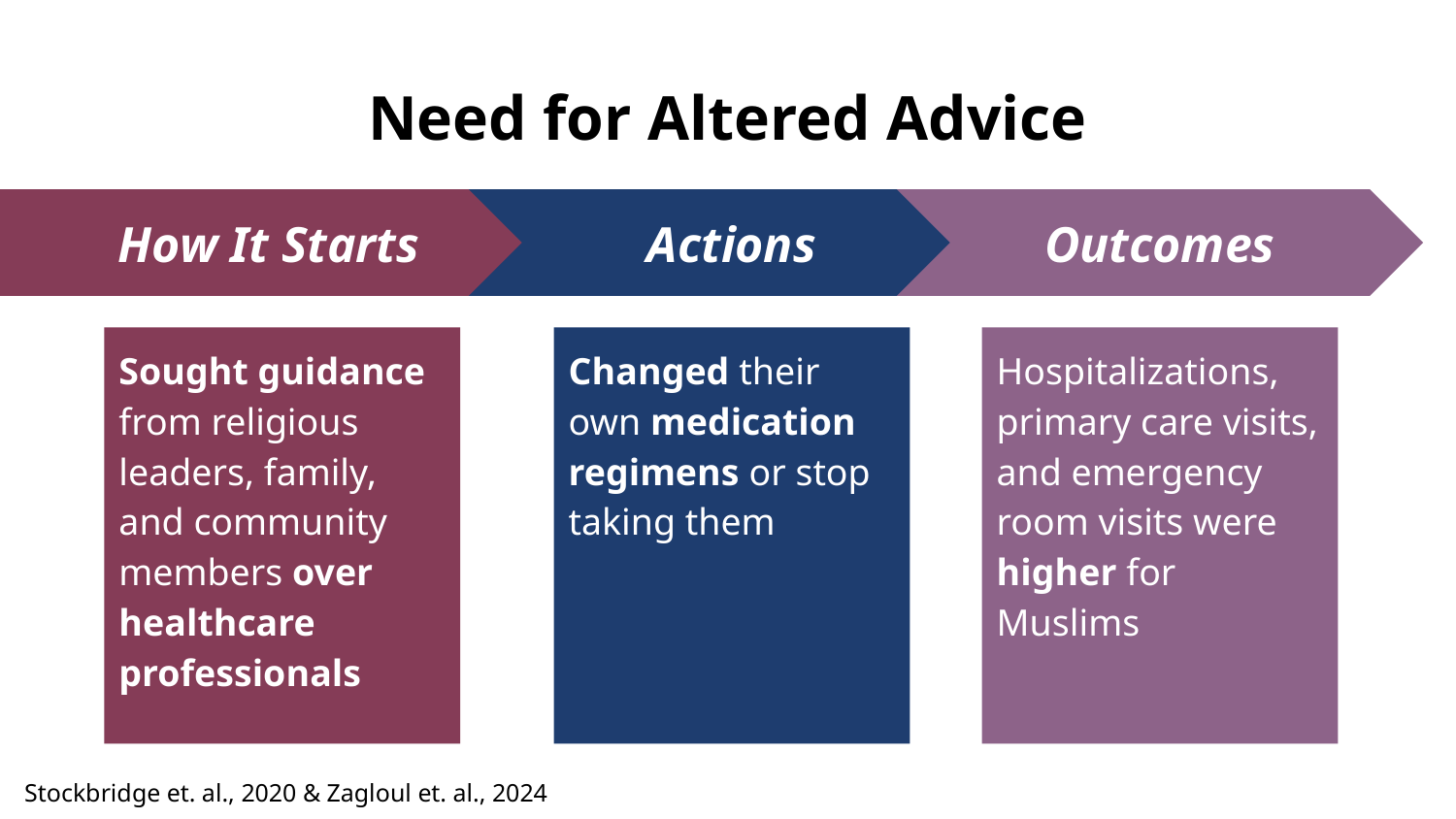

# Need for Altered Advice
Actions
Changed their own medication regimens or stop taking them
Outcomes
Hospitalizations, primary care visits, and emergency room visits were higher for Muslims
How It Starts
Sought guidance from religious leaders, family, and community members over healthcare professionals
Stockbridge et. al., 2020 & Zagloul et. al., 2024

## Slide 11
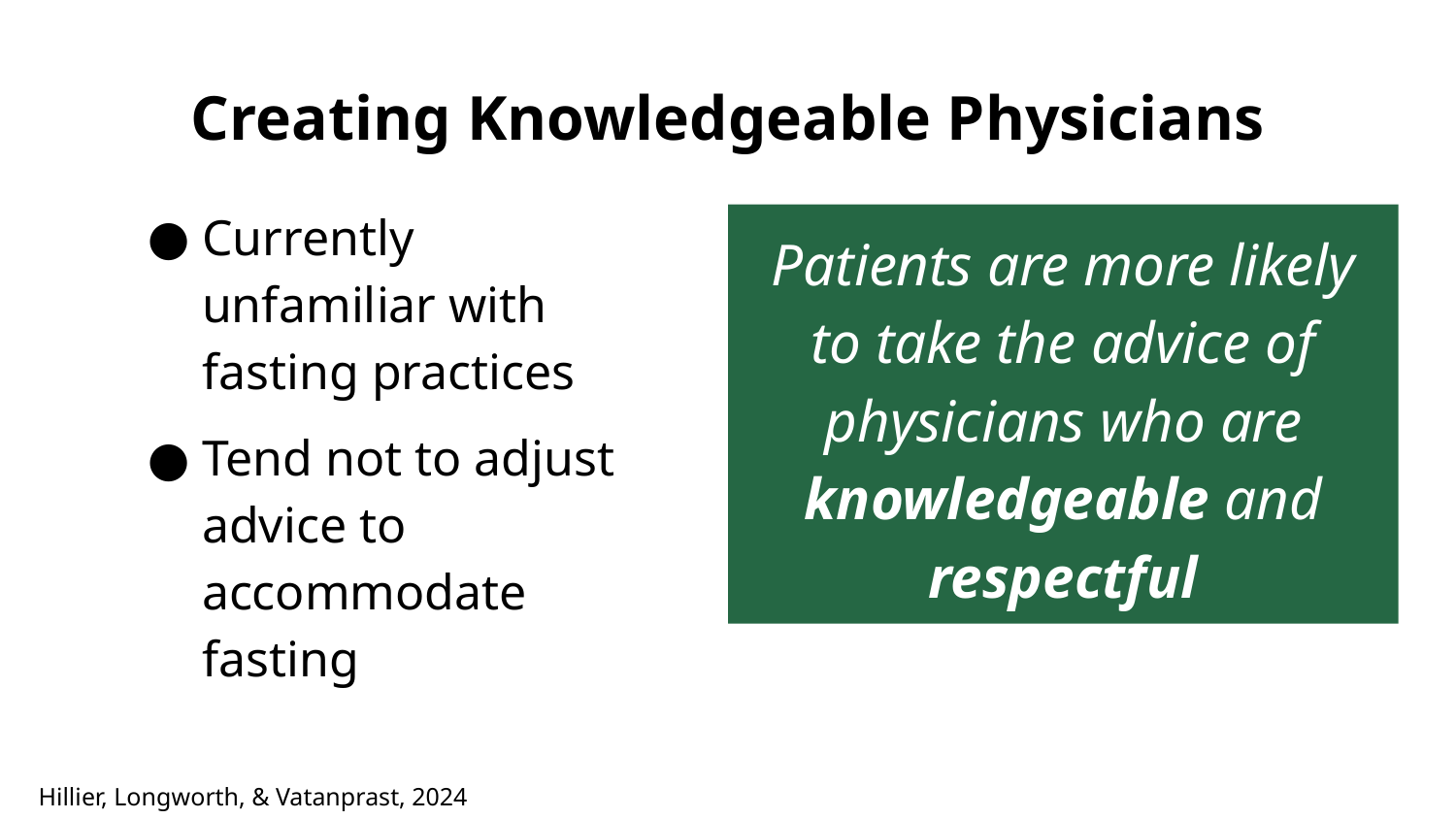

# Creating Knowledgeable Physicians
Currently unfamiliar with fasting practices
Tend not to adjust advice to accommodate fasting
Patients are more likely to take the advice of physicians who are knowledgeable and respectful
Hillier, Longworth, & Vatanprast, 2024

## Slide 12
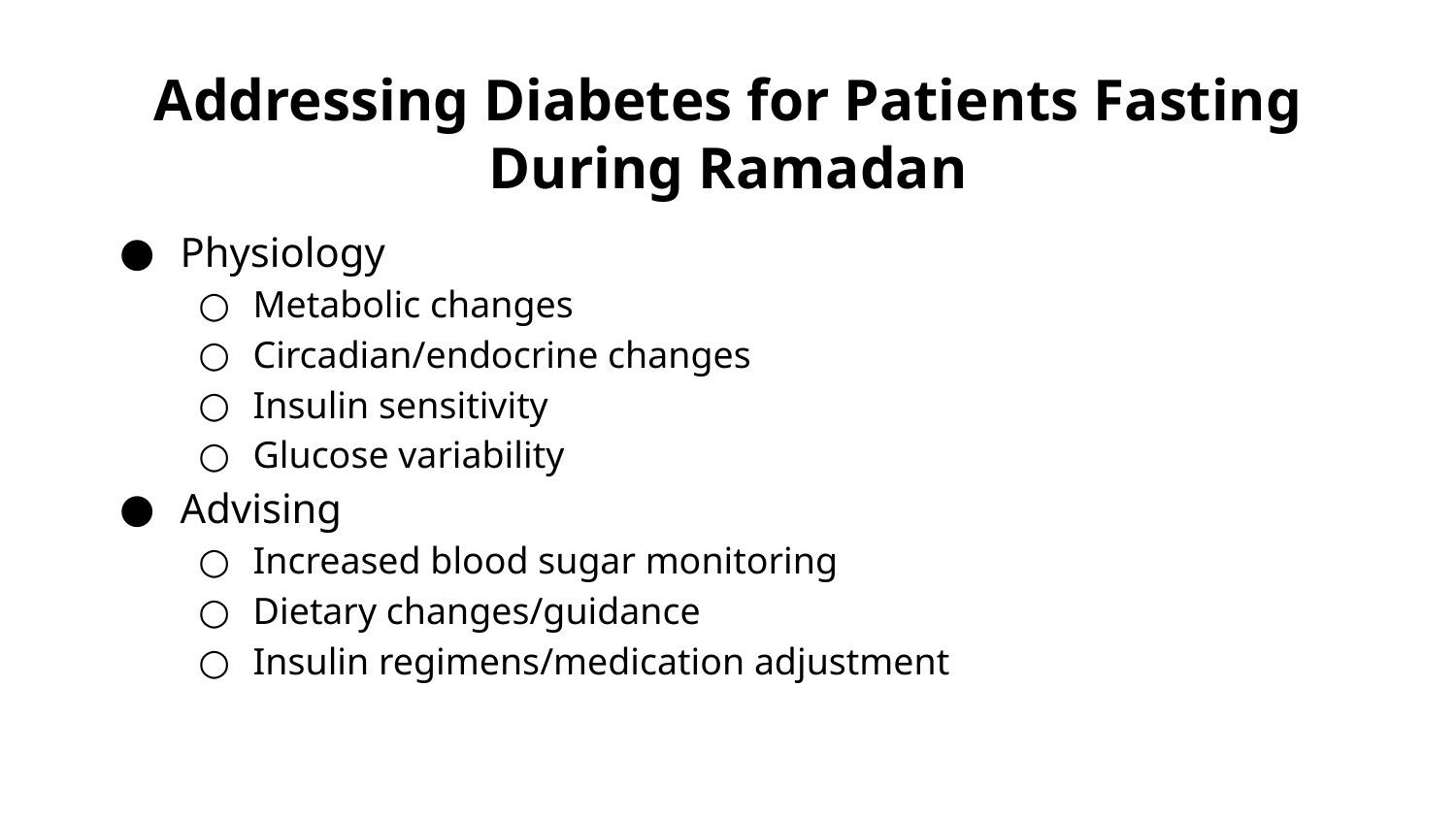

# Addressing Diabetes for Patients Fasting During Ramadan
Physiology
Metabolic changes
Circadian/endocrine changes
Insulin sensitivity
Glucose variability
Advising
Increased blood sugar monitoring
Dietary changes/guidance
Insulin regimens/medication adjustment

## Slide 13
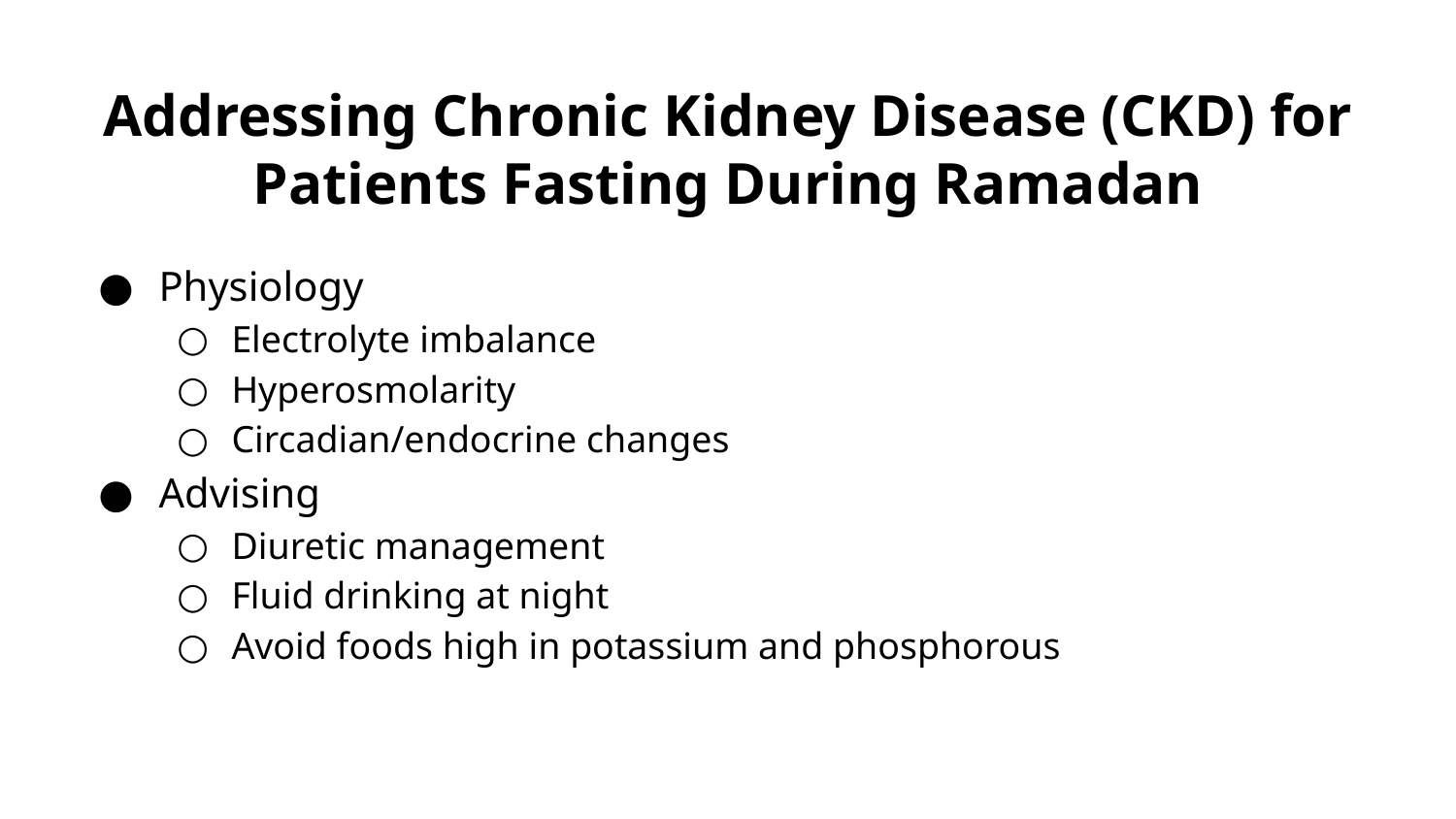

# Addressing Chronic Kidney Disease (CKD) for Patients Fasting During Ramadan
Physiology
Electrolyte imbalance
Hyperosmolarity
Circadian/endocrine changes
Advising
Diuretic management
Fluid drinking at night
Avoid foods high in potassium and phosphorous

## Slide 14
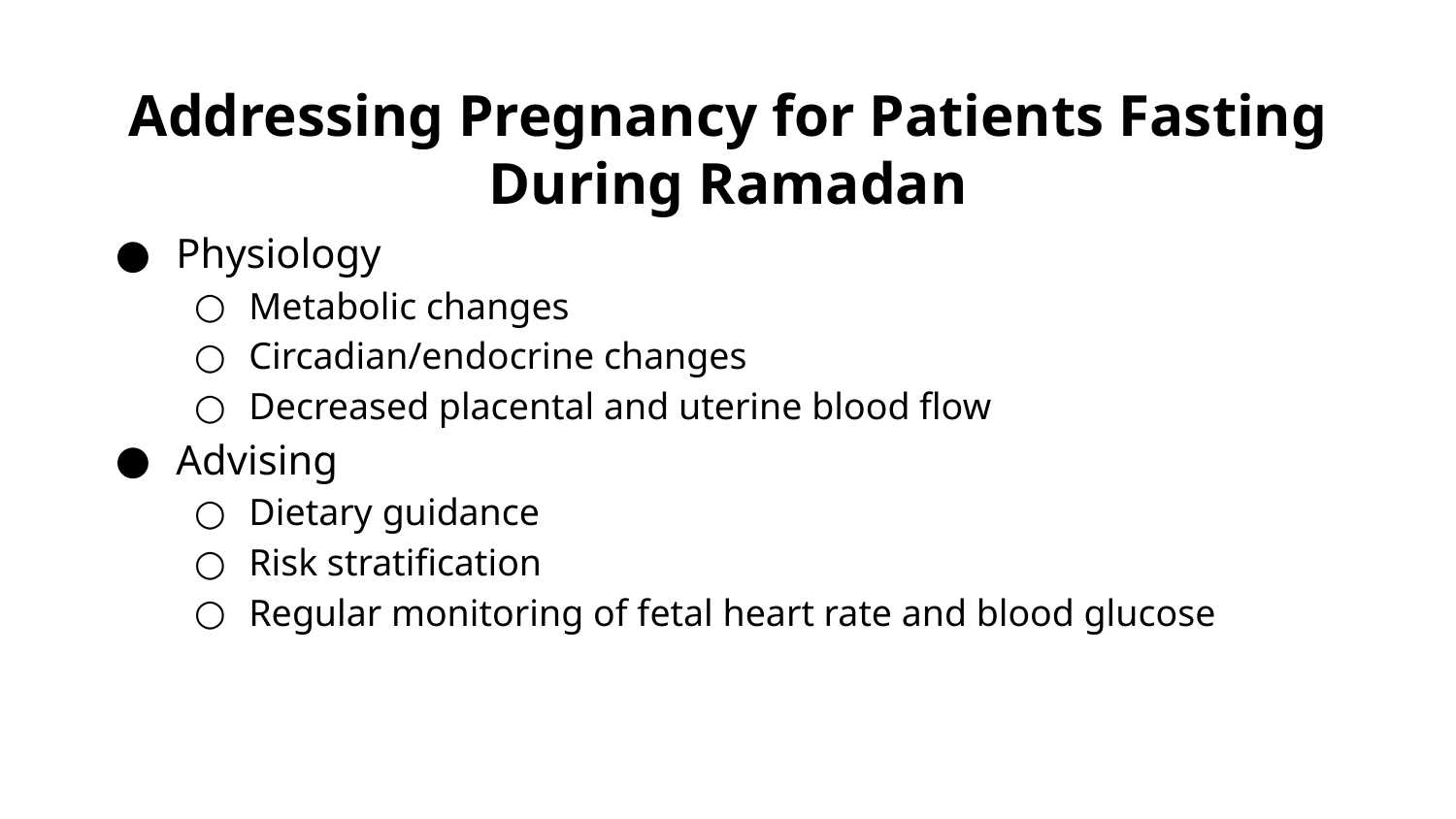

# Addressing Pregnancy for Patients Fasting During Ramadan
Physiology
Metabolic changes
Circadian/endocrine changes
Decreased placental and uterine blood flow
Advising
Dietary guidance
Risk stratification
Regular monitoring of fetal heart rate and blood glucose

## Slide 15
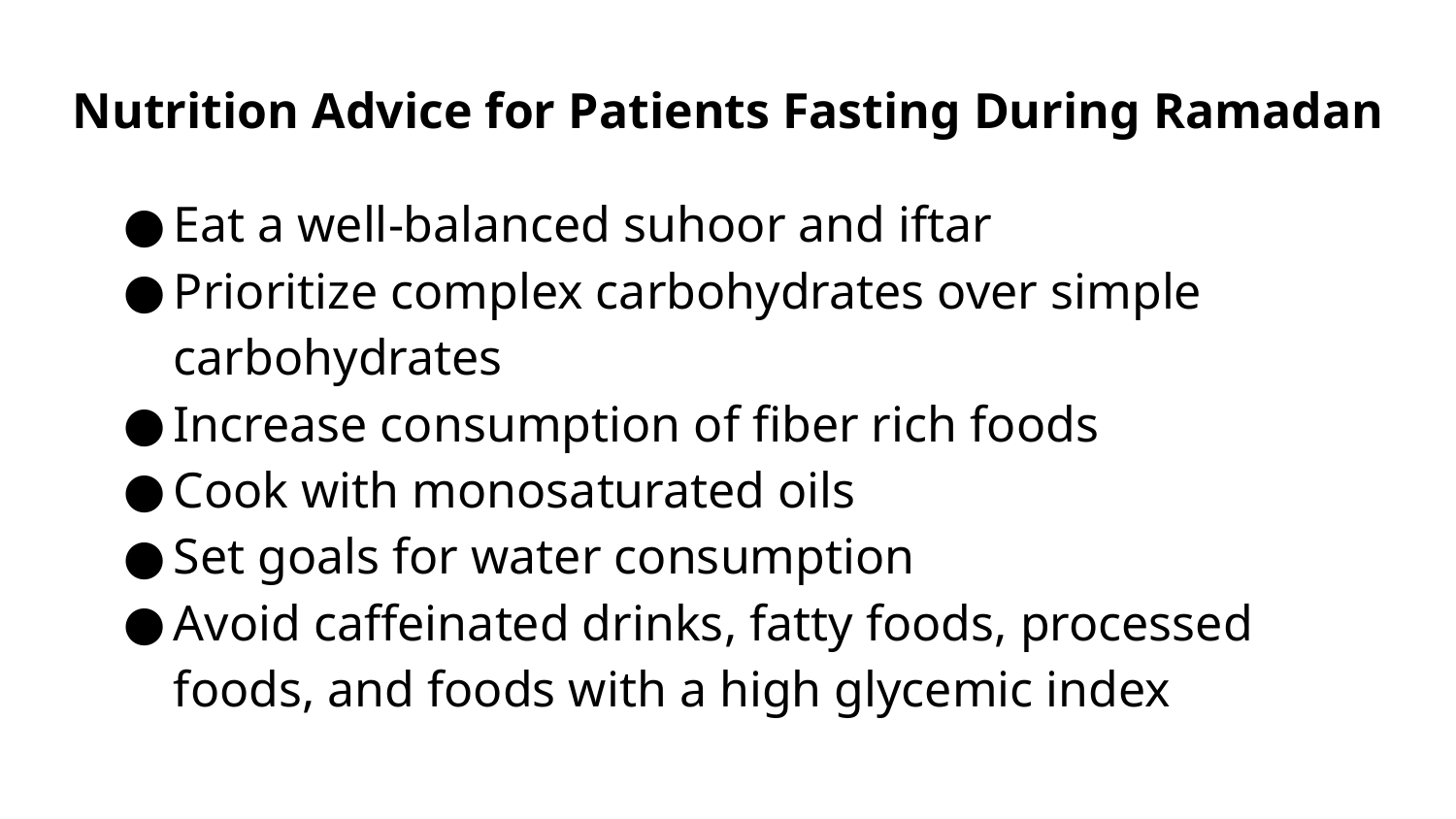

# Nutrition Advice for Patients Fasting During Ramadan
Eat a well-balanced suhoor and iftar
Prioritize complex carbohydrates over simple carbohydrates
Increase consumption of fiber rich foods
Cook with monosaturated oils
Set goals for water consumption
Avoid caffeinated drinks, fatty foods, processed foods, and foods with a high glycemic index

## Slide 16
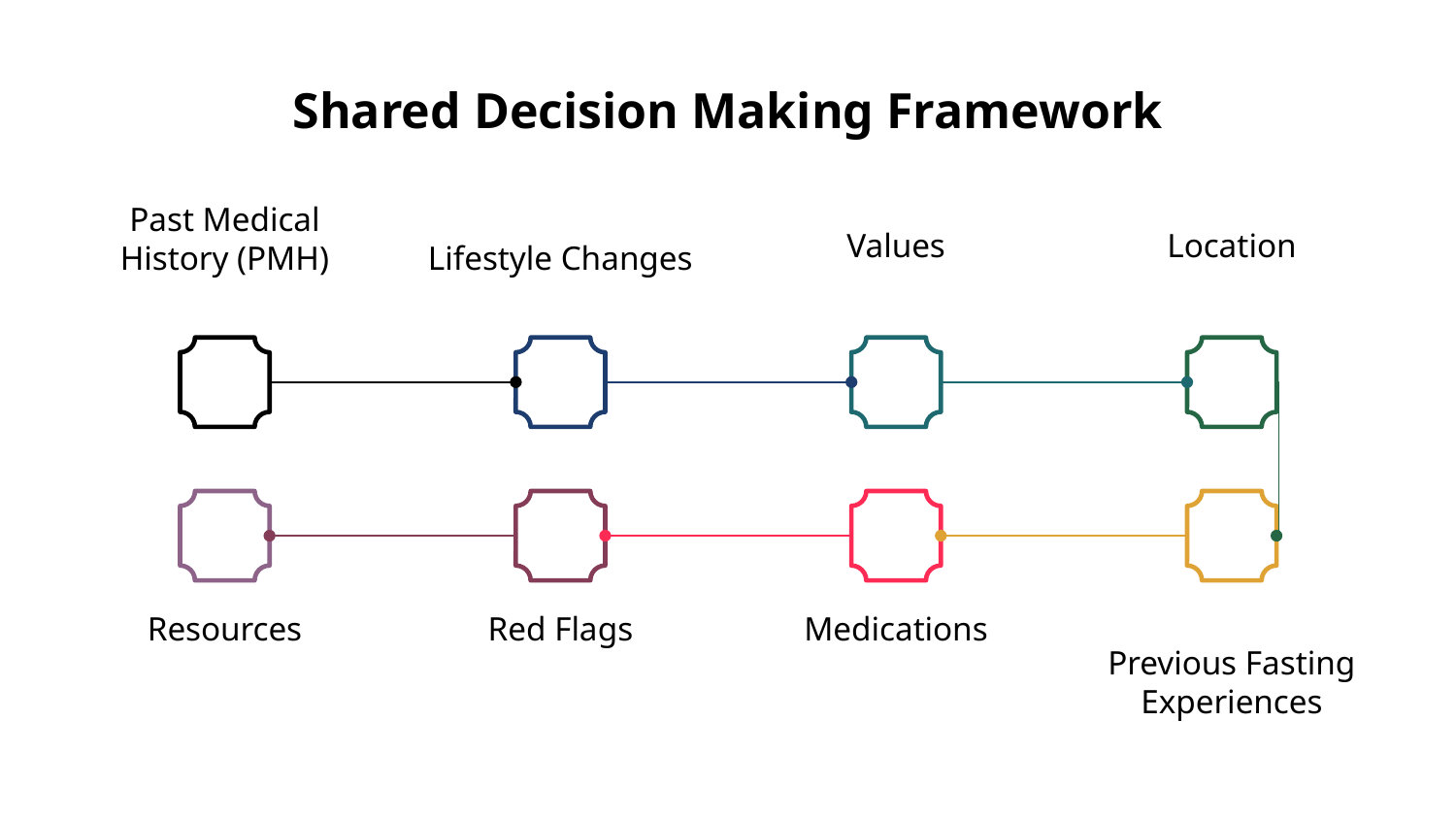

# Shared Decision Making Framework
Values
Location
Past Medical History (PMH)
Lifestyle Changes
Resources
Red Flags
Medications
Previous Fasting Experiences

## Slide 17
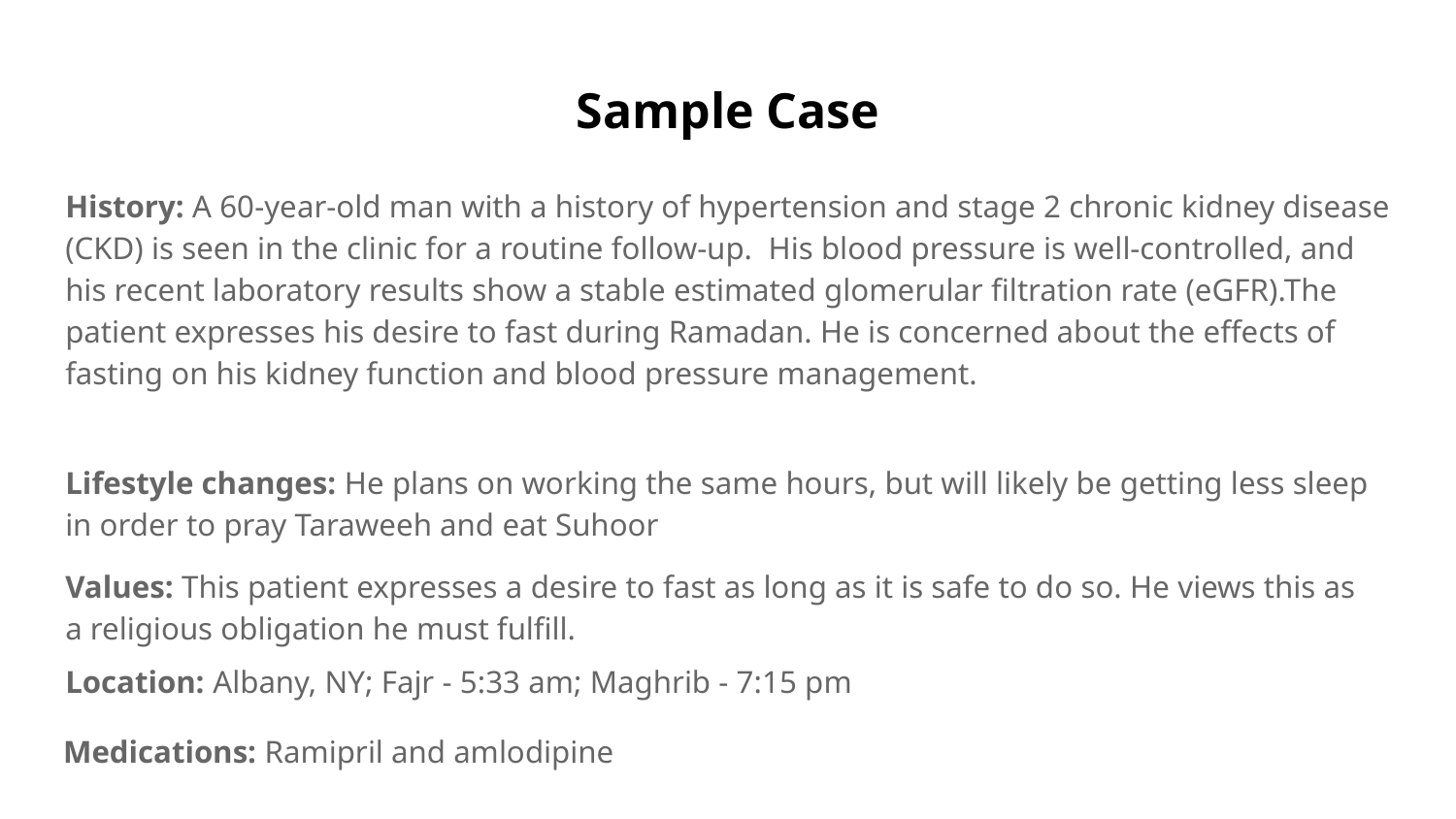

# Sample Case
History: A 60-year-old man with a history of hypertension and stage 2 chronic kidney disease (CKD) is seen in the clinic for a routine follow-up. His blood pressure is well-controlled, and his recent laboratory results show a stable estimated glomerular filtration rate (eGFR).The patient expresses his desire to fast during Ramadan. He is concerned about the effects of fasting on his kidney function and blood pressure management.
Lifestyle changes: He plans on working the same hours, but will likely be getting less sleep in order to pray Taraweeh and eat Suhoor
Values: This patient expresses a desire to fast as long as it is safe to do so. He views this as a religious obligation he must fulfill.
Location: Albany, NY; Fajr - 5:33 am; Maghrib - 7:15 pm
Medications: Ramipril and amlodipine

## Slide 18
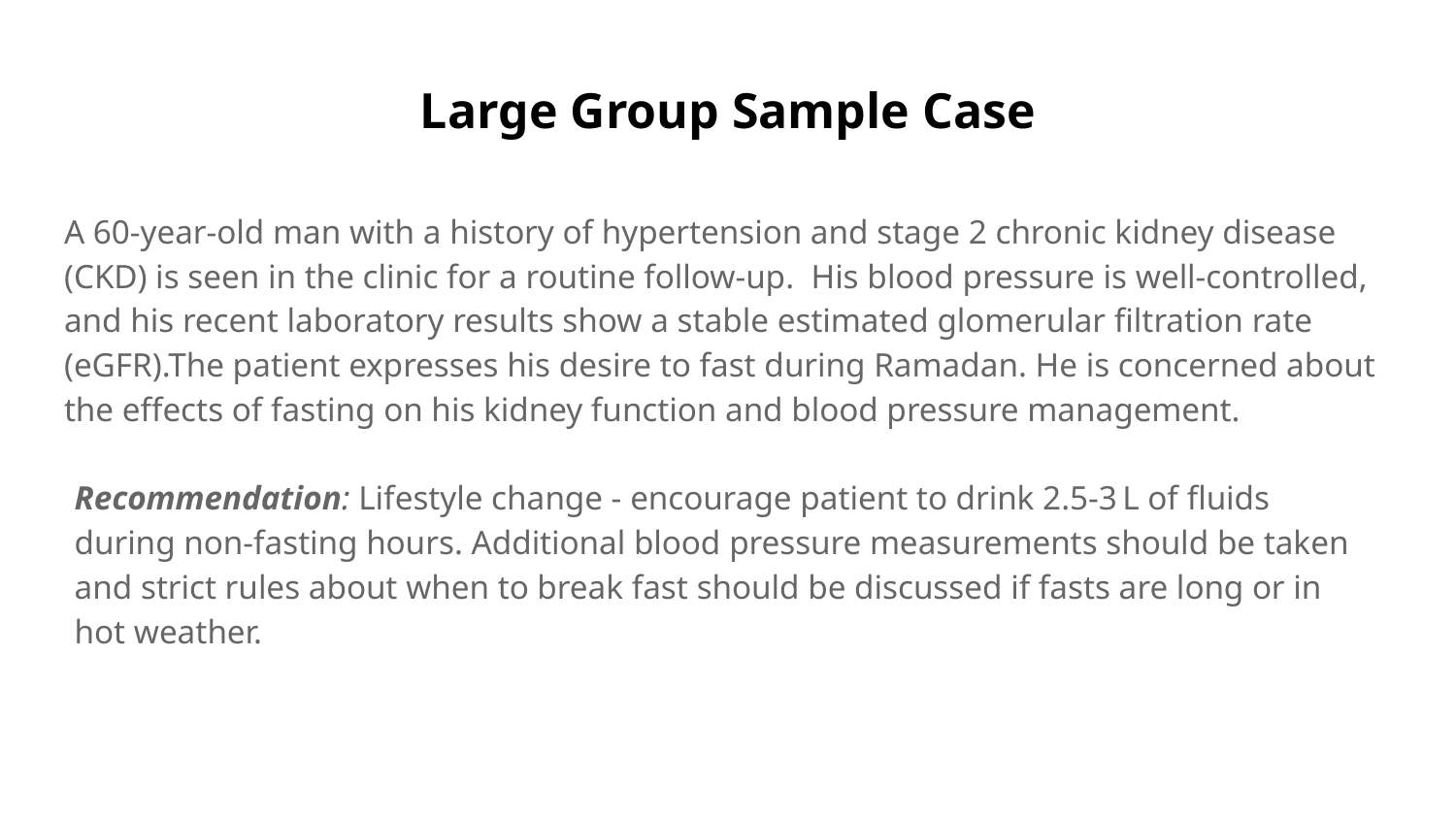

# Large Group Sample Case
A 60-year-old man with a history of hypertension and stage 2 chronic kidney disease (CKD) is seen in the clinic for a routine follow-up. His blood pressure is well-controlled, and his recent laboratory results show a stable estimated glomerular filtration rate (eGFR).The patient expresses his desire to fast during Ramadan. He is concerned about the effects of fasting on his kidney function and blood pressure management.
Recommendation: Lifestyle change - encourage patient to drink 2.5-3 L of fluids during non-fasting hours. Additional blood pressure measurements should be taken and strict rules about when to break fast should be discussed if fasts are long or in hot weather.

## Slide 19
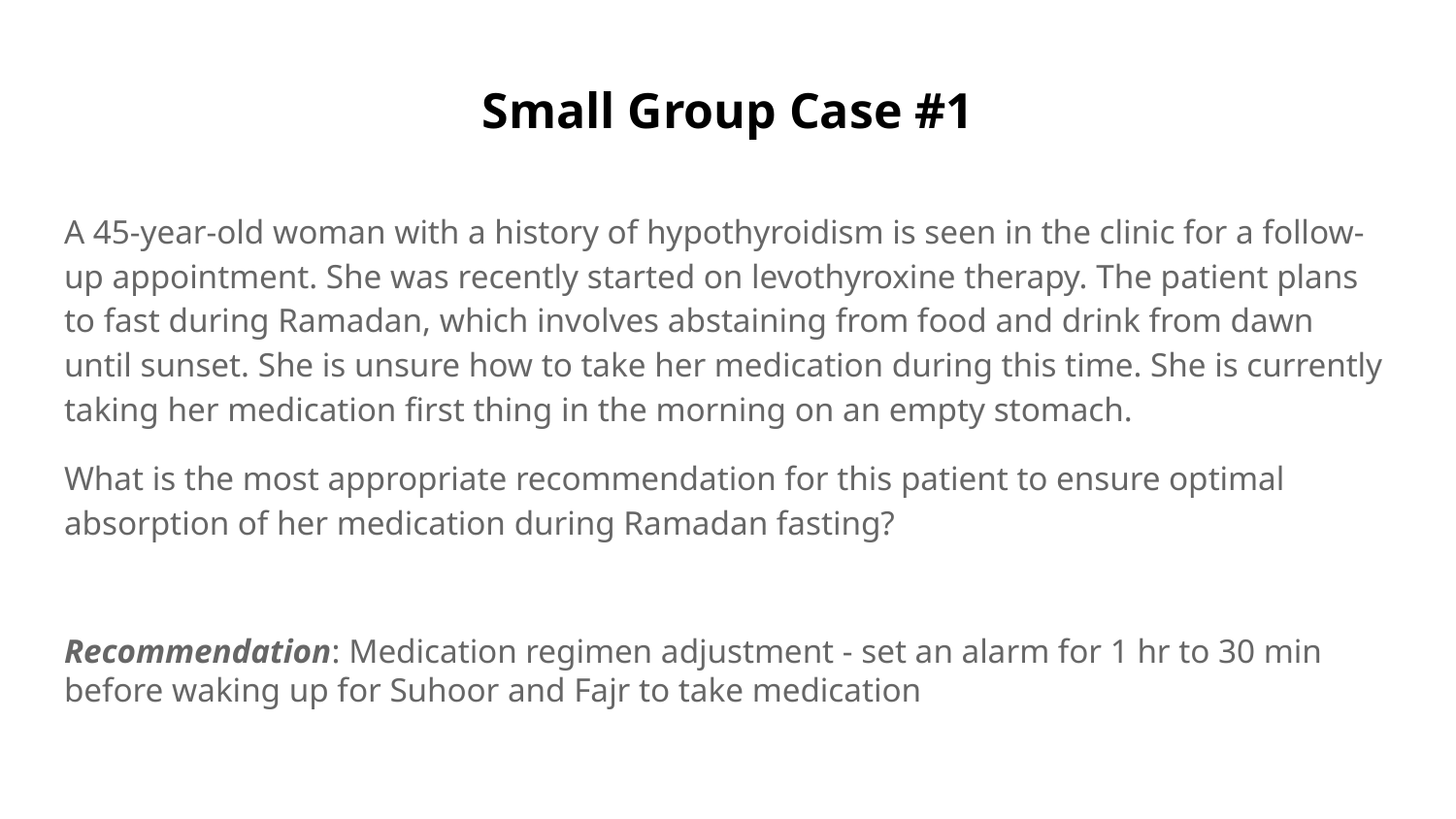

# Small Group Case #1
A 45-year-old woman with a history of hypothyroidism is seen in the clinic for a follow-up appointment. She was recently started on levothyroxine therapy. The patient plans to fast during Ramadan, which involves abstaining from food and drink from dawn until sunset. She is unsure how to take her medication during this time. She is currently taking her medication first thing in the morning on an empty stomach.
What is the most appropriate recommendation for this patient to ensure optimal absorption of her medication during Ramadan fasting?
Recommendation: Medication regimen adjustment - set an alarm for 1 hr to 30 min before waking up for Suhoor and Fajr to take medication

## Slide 20
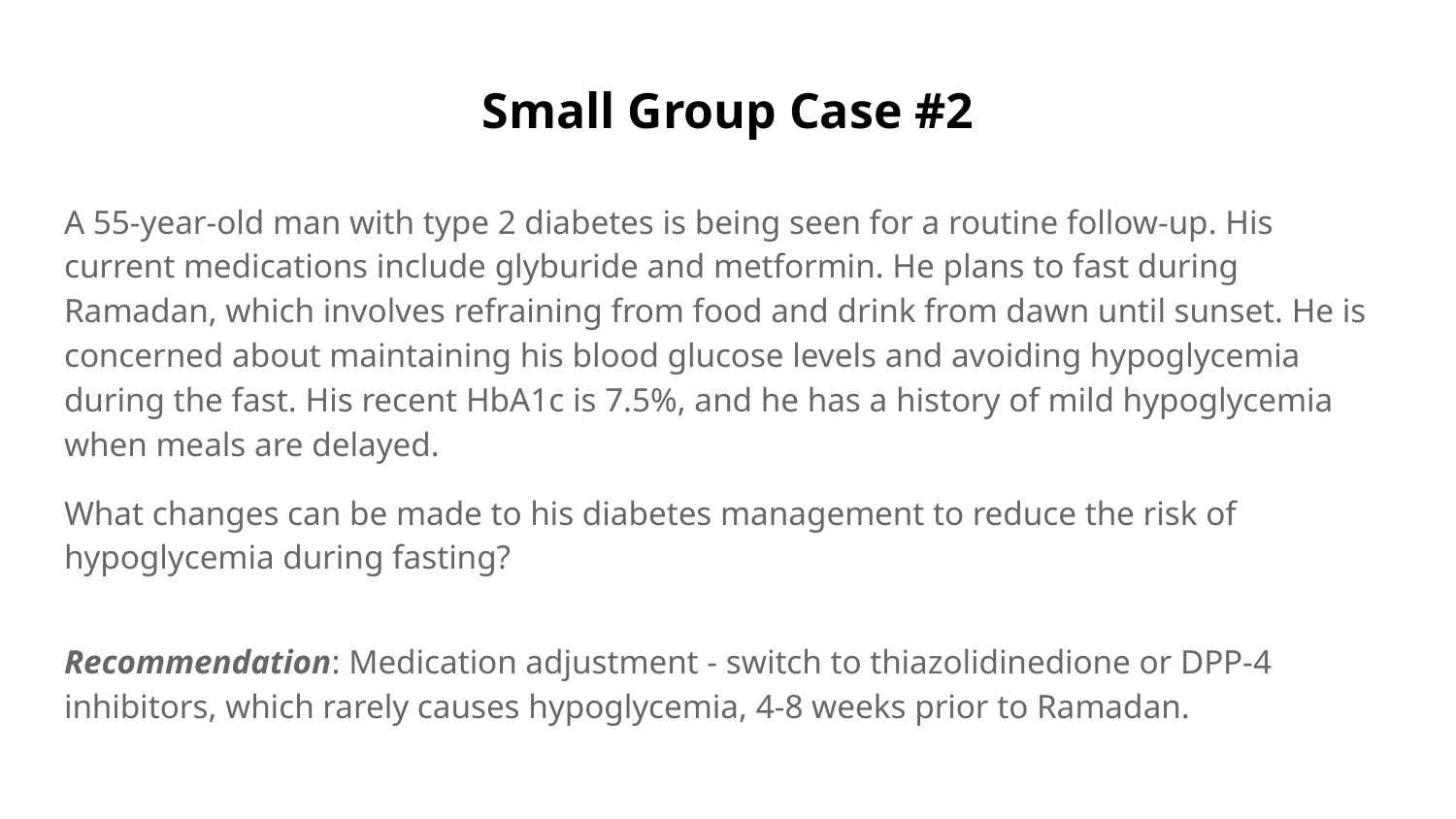

# Small Group Case #2
A 55-year-old man with type 2 diabetes is being seen for a routine follow-up. His current medications include glyburide and metformin. He plans to fast during Ramadan, which involves refraining from food and drink from dawn until sunset. He is concerned about maintaining his blood glucose levels and avoiding hypoglycemia during the fast. His recent HbA1c is 7.5%, and he has a history of mild hypoglycemia when meals are delayed.
What changes can be made to his diabetes management to reduce the risk of hypoglycemia during fasting?
Recommendation: Medication adjustment - switch to thiazolidinedione or DPP-4 inhibitors, which rarely causes hypoglycemia, 4-8 weeks prior to Ramadan.

## Slide 21
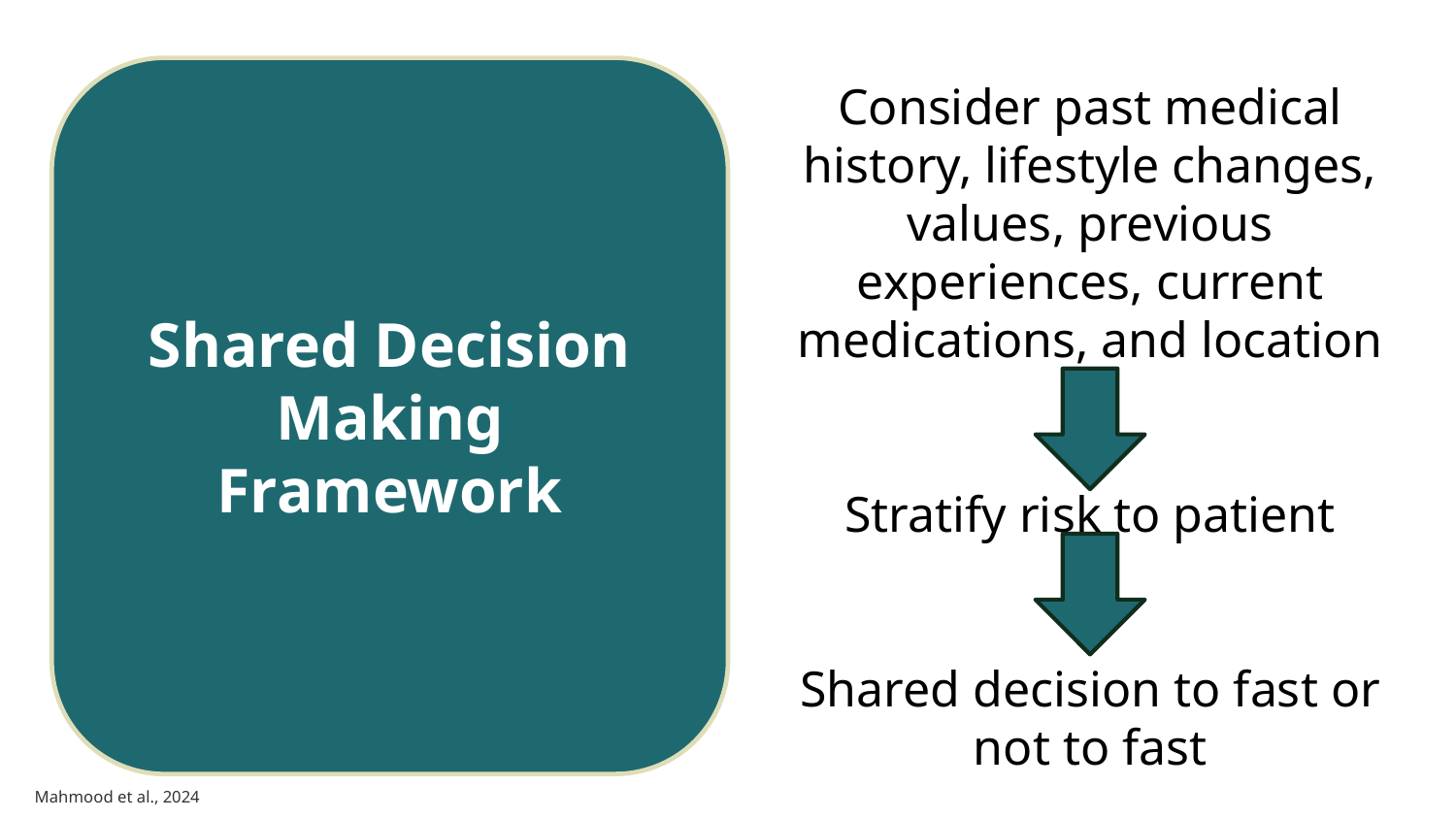

Shared Decision Making Framework
Consider past medical history, lifestyle changes, values, previous experiences, current medications, and location
Stratify risk to patient
Shared decision to fast or not to fast
 Mahmood et al., 2024

## Slide 22
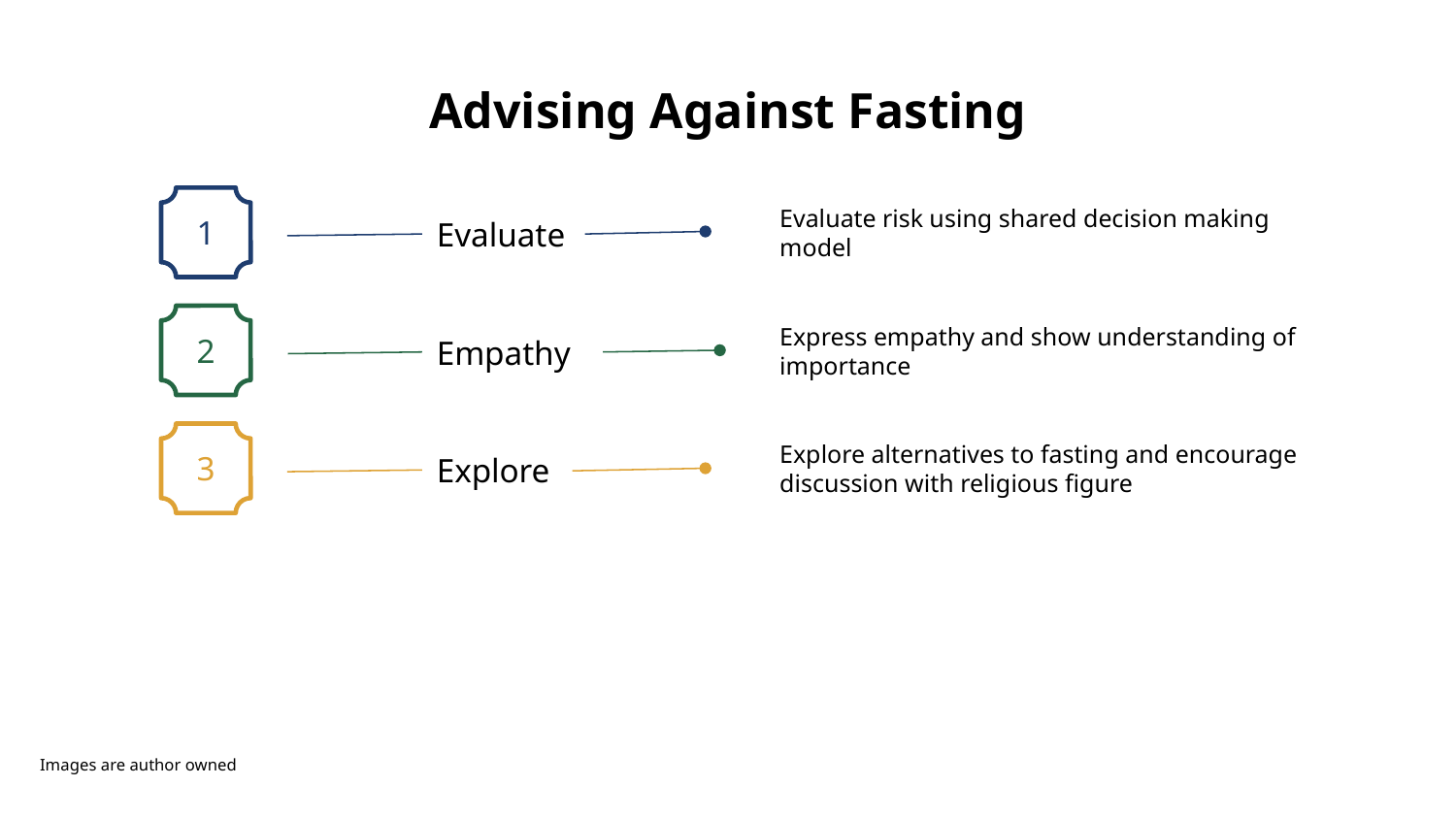

# Advising Against Fasting
1
Evaluate risk using shared decision making model
Evaluate
2
Express empathy and show understanding of importance
Empathy
3
Explore alternatives to fasting and encourage discussion with religious figure
Explore
Images are author owned

## Slide 23
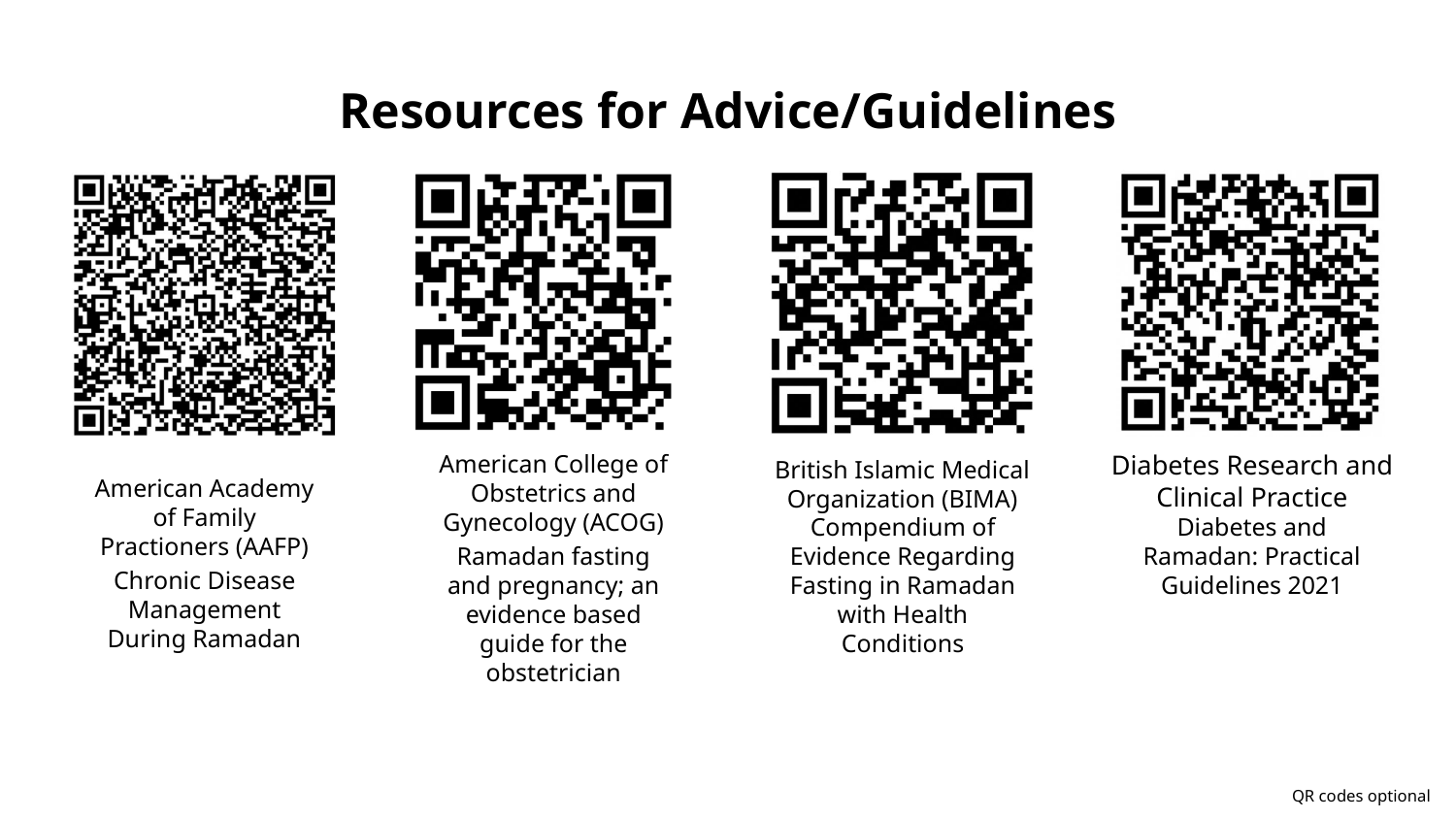

# Resources for Advice/Guidelines
Diabetes Research and Clinical Practice
British Islamic Medical Organization (BIMA)
American College of Obstetrics and Gynecology (ACOG)
Compendium of Evidence Regarding Fasting in Ramadan with Health Conditions
Diabetes and Ramadan: Practical Guidelines 2021
American Academy of Family Practioners (AAFP)
Ramadan fasting and pregnancy; an evidence based guide for the obstetrician
Chronic Disease Management During Ramadan
QR codes optional

## Slide 24
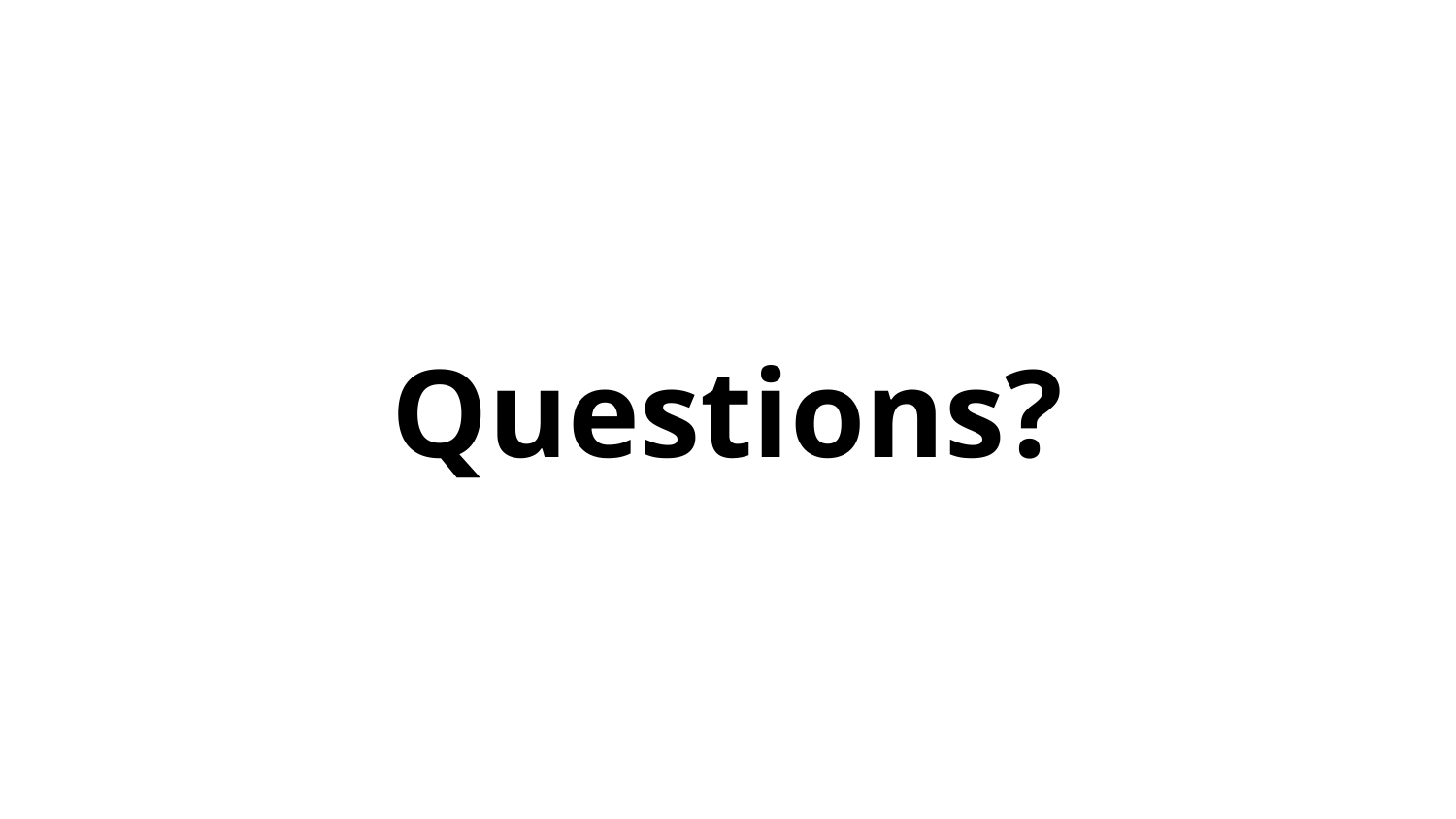

# Questions?

## Slide 25
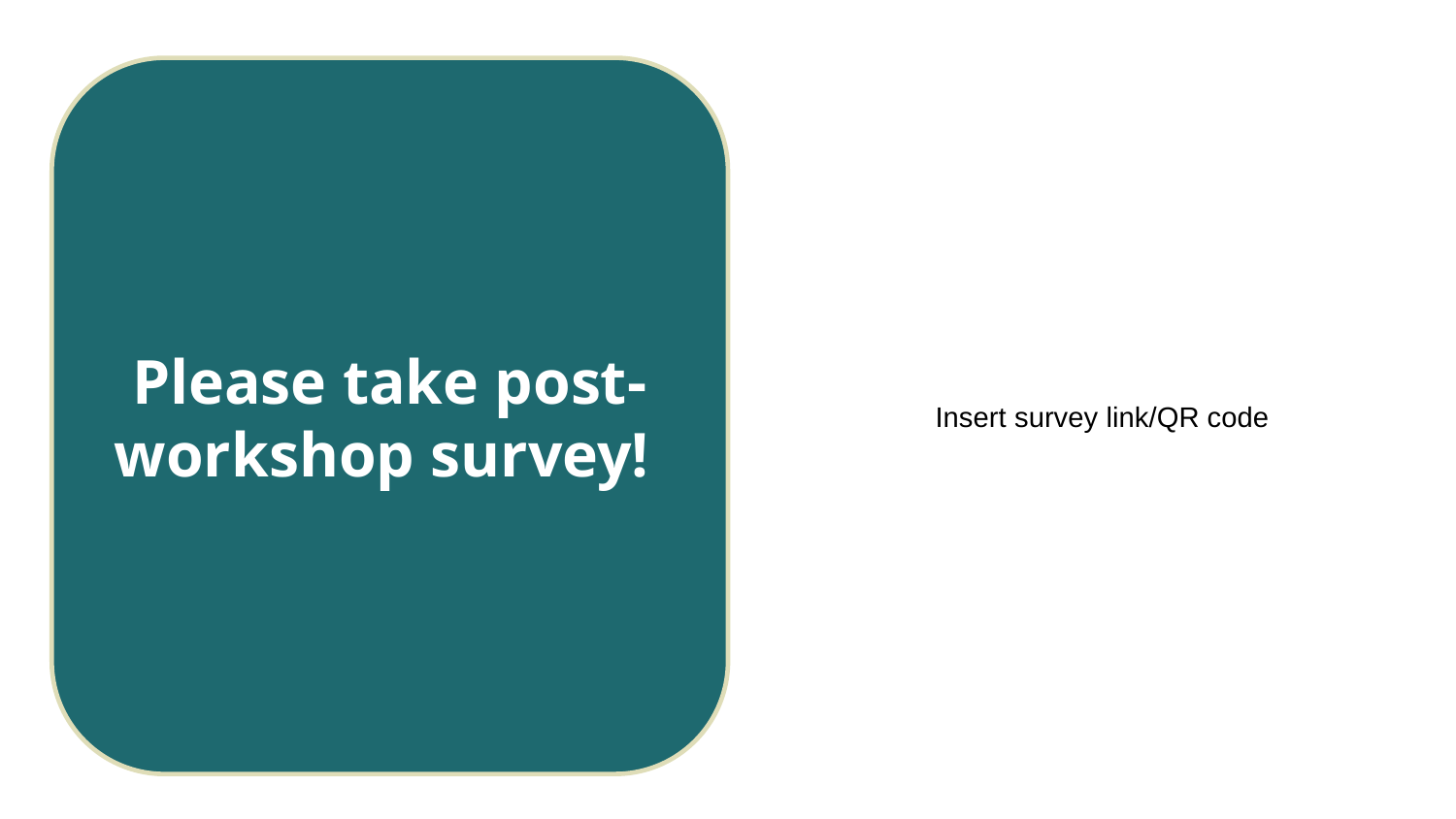

Please take post-workshop survey!
Insert survey link/QR code

## Slide 26
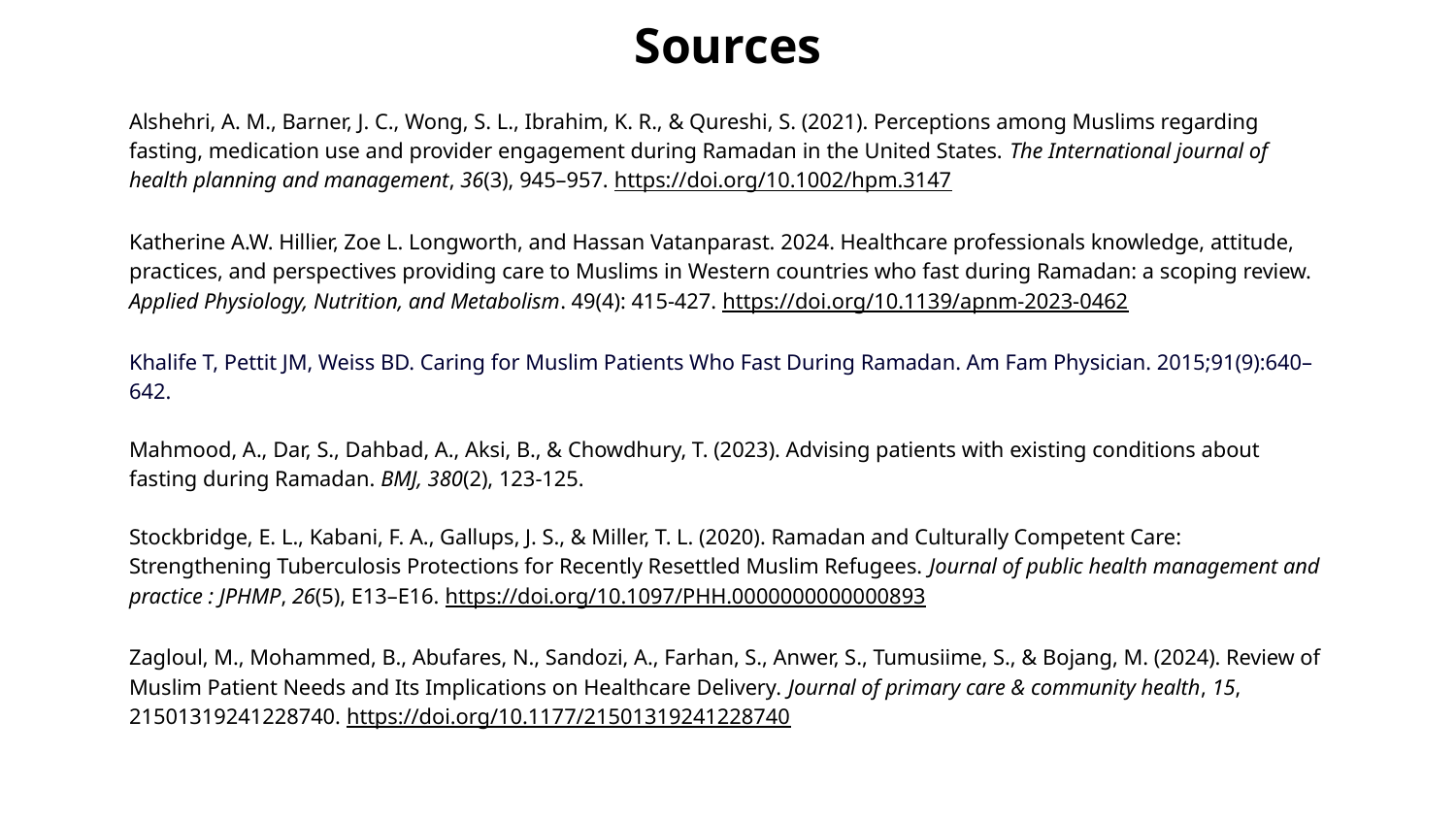

# Sources
Alshehri, A. M., Barner, J. C., Wong, S. L., Ibrahim, K. R., & Qureshi, S. (2021). Perceptions among Muslims regarding fasting, medication use and provider engagement during Ramadan in the United States. The International journal of health planning and management, 36(3), 945–957. https://doi.org/10.1002/hpm.3147
Katherine A.W. Hillier, Zoe L. Longworth, and Hassan Vatanparast. 2024. Healthcare professionals knowledge, attitude, practices, and perspectives providing care to Muslims in Western countries who fast during Ramadan: a scoping review. Applied Physiology, Nutrition, and Metabolism. 49(4): 415-427. https://doi.org/10.1139/apnm-2023-0462
Khalife T, Pettit JM, Weiss BD. Caring for Muslim Patients Who Fast During Ramadan. Am Fam Physician. 2015;91(9):640–642.
Mahmood, A., Dar, S., Dahbad, A., Aksi, B., & Chowdhury, T. (2023). Advising patients with existing conditions about fasting during Ramadan. BMJ, 380(2), 123-125.
Stockbridge, E. L., Kabani, F. A., Gallups, J. S., & Miller, T. L. (2020). Ramadan and Culturally Competent Care: Strengthening Tuberculosis Protections for Recently Resettled Muslim Refugees. Journal of public health management and practice : JPHMP, 26(5), E13–E16. https://doi.org/10.1097/PHH.0000000000000893
Zagloul, M., Mohammed, B., Abufares, N., Sandozi, A., Farhan, S., Anwer, S., Tumusiime, S., & Bojang, M. (2024). Review of Muslim Patient Needs and Its Implications on Healthcare Delivery. Journal of primary care & community health, 15, 21501319241228740. https://doi.org/10.1177/21501319241228740

## Slide 27
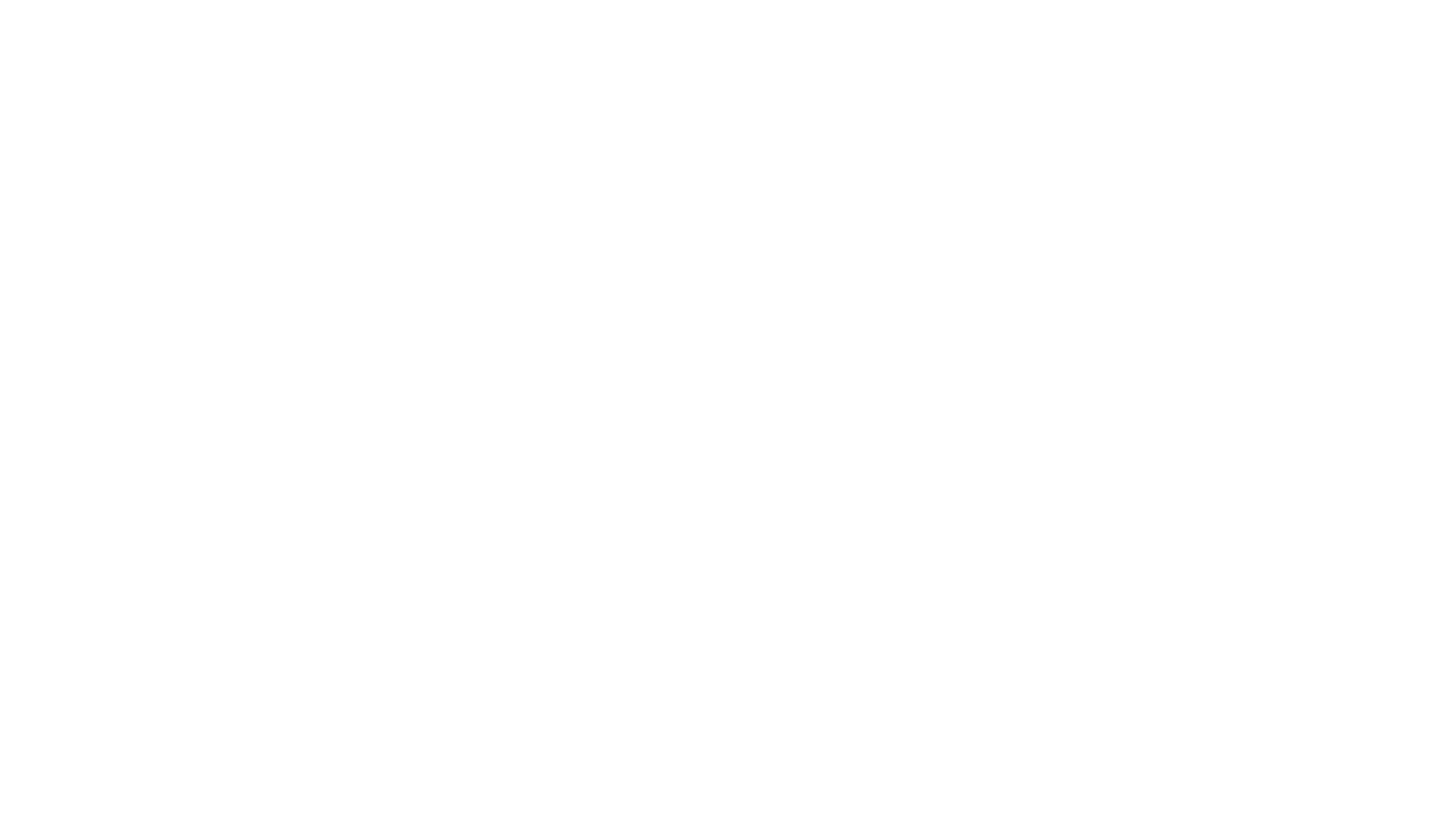

Supplement: Supplementary file 1 — Facilitator Guide.docxRamadan and the Fasting Patient Module.pptxPreworkshop and Postworkshop Survey.docx [file mep_2374-8265.11614-s001.zip › B. Ramadan and the Fasting Patient Module.pptx]
