# Supplementary material for: Clinical Considerations While Providing Care for Patients During Ramadan: A Framework for Health Care Professionals
Source: MedEdPORTAL. 2026 Jun 25;22:11614. doi: 10.15766/mep_2374-8265.11614 (PMC13294187; doi:10.15766/mep_2374-8265.11614)
Supplement: Supplementary file 1 — Facilitator Guide.docxRamadan and the Fasting Patient Module.pptxPreworkshop and Postworkshop Survey.docx [file mep_2374-8265.11614-s001.zip › C. Preworkshop and Postworkshop Survey.docx]

Pre-Workshop/Post-Workshop Survey

(Administer prior to the workshop beginning and repeat immediately following)

Demographic

- What is your gender?
  - Man
  - Woman
  - Non-Binary
  - Other
- What is your level of training?
  - Attending
  - Fellow
  - Resident
  - MS4
  - MS3
  - MS2
  - MS1
- What is your race/ethnicity?
  - White/Caucasian
  - Black/African American
  - Arab/Arab American
  - South Asian/South Asian American
  - Asian/Asian American
  - Indigenous American/Alaskan Native
  - Hispanic/Latino
  - Other
- What is your religion?
  - Christianity
  - Judaism
  - Islam
  - Hinduism
  - Agnostic
  - Atheist
  - Other
  - None
- How would you rate your religiosity?
  - Very religious
  - Moderately religious
  - Somewhat religious
  - Not religious at all
  - Other
  - Prefer not to answer
- Have you ever participated in a fast for Ramadan before? Yes
  - No

Knowledge

- How much exposure have you had working with Muslim patients?
  - None
  - A little
  - A moderate amount
  - A lot
  - A great deal
- (LO#1) True or False: People fasting for Ramadan must abstain from eating or drinking from sunrise to sunset.
  - True
  - False
- (LO#1) True or False: People fasting for Ramadan must abstain from drinking water from sunrise to sunset.
  - True
  - False
- (LO#1) True or False: People fasting for Ramadan must abstain from taking oral medications or IV fluids from sunrise to sunset.
  - True
  - False
- (LO#1) True or False: Every practicing Muslim is required to fast during Ramadan, regardless of chronic conditions.
  - True
  - False
- (LO#1) True or False: Ramadan takes place at the same time each year.
  - True
  - False

Comfort – Please state your agreement with the following statements.

- (LO #3) I feel comfortable asking patients if they are fasting for Ramadan (either directly or indirectly).
  - Disagree
  - Somewhat disagree
  - Neutral
  - Somewhat agree
  - Agree
- (LO #4) I am comfortable providing care to patients who are fasting for Ramadan.
  - Disagree
  - Somewhat disagree
  - Neutral
  - Somewhat agree
  - Agree
- (LO #4) I am comfortable providing care to patients with underlying conditions who are fasting for Ramadan.
  - Disagree
  - Somewhat disagree
  - Neutral
  - Somewhat agree
  - Agree
- (LO #3) I am comfortable obtaining a relevant social and medical history from patients who are fasting for Ramadan.
  - Disagree
  - Somewhat disagree
  - Neutral
  - Somewhat agree
  - Agree
- (LO #4) I am comfortable adjusting medications/medication use practices for patients who are fasting for Ramadan.
  - Disagree
  - Somewhat disagree
  - Neutral
  - Somewhat agree
  - Agree
- (LO #2) I feel comfortable advocating on behalf of a patient who is fasting for Ramadan to another healthcare provider who is unaware of the patient’s current practices.
  - Disagree
  - Somewhat disagree
  - Neutral
  - Somewhat agree
  - Agree
- (LO #2) Understanding a patient’s religious beliefs and values is an important factor in providing care and meeting their healthcare needs.
  - Disagree
  - Somewhat disagree
  - Neutral
  - Somewhat agree
  - Agree
- (LO#2) Fasting for Ramadan may impact the way that I advise patients to manage their health.
  - Disagree
  - Somewhat disagree
  - Neutral
  - Somewhat agree
  - Agree
- (LO #4) I can find resources that will help me understand how to effectively manage patients fasting for Ramadan.
  - Disagree
  - Somewhat disagree
  - Neutral
  - Somewhat agree
  - Agree
- (LO #2) I understand how religious practices for Ramadan can impact patient health.
  - Disagree
  - Somewhat disagree
  - Neutral
  - Somewhat agree
  - Agree
