# Supplementary material for: Clinical Considerations While Providing Care for Patients During Ramadan: A Framework for Health Care Professionals
Source: MedEdPORTAL. 2026 Jun 25;22:11614. doi: 10.15766/mep_2374-8265.11614 (PMC13294187; doi:10.15766/mep_2374-8265.11614)
Supplement: Supplementary file 1 — Facilitator Guide.docxRamadan and the Fasting Patient Module.pptxPreworkshop and Postworkshop Survey.docx [file mep_2374-8265.11614-s001.zip › _Educational Summary Report_11614.pdf]

MedEdPORTAL
THE AAMC JOURNAL OF TEACHING AND LEARNING RESOURCES

Original Publication

Open Access

Clinical Considerations While Providing Care for Patients During Ramadan: A Framework for Health Care Professionals

Summer Khan*, Zaina Siraj, MD, Ashar Ata, MPH, PhD, Nyla Azam, MD

*Corresponding author: khans25@amc.edu

Abstract

Introduction: Fasting during Ramadan is an important religious obligation for Muslims, yet many health care providers are unaware of what it entails. Consequently, patients with chronic diseases often rely on community advice to manage their conditions while fasting. This has contributed to increased hospitalizations, primary care visits, and emergency department utilization during Ramadan. This workshop was created to address knowledge gaps and support providers in counseling patients on disease management while fasting. **Methods:** This 1-hour workshop outlined the significance of fasting during Ramadan and common medical conditions affected by fasting, and introduced a framework for tailoring medical advice to fasting patients. Pre- and postworkshop surveys were administered to graduate-level medical professionals to assess changes in participants' knowledge and comfort in addressing Ramadan-specific care. **Results:** Fifty-four matched pre- and postworkshop survey responses were collected from participants, including medical students, residents, attending physicians, nurses, and other health care professionals. McNemar's chi square test demonstrated significant increases in participants' knowledge of religious practices during Ramadan ($P < .001$), understanding of how these practices can impact health ($P < .05$), and comfort in offering adapted medical advice ($P < .05$). **Discussion:** Many participants were aware that fasting during Ramadan occurs; however, few recognized a need to modify medical recommendations. Following this workshop, participants reported a statistically significant greater preparedness to discuss disease management with fasting patients and offer culturally sensitive guidance. Supporting patients to pursue customs and traditions that are important to them can strengthen trust in the patient–provider relationship and improve care delivery.

Keywords
Islam, Ramadan, Allyship, Fasting, Religion, Cultural Humility, Arab Health, Muslim Health

Educational Objectives

By the end of this activity, learners will be able to:

1. Identify the key elements of Ramadan and its religious importance to understand the values of Muslim patients who wish to fast.
2. Recognize the clinical implications of fasting for patients with chronic conditions and the need for altered medical advising to control conditions during Ramadan.
3. Discuss a framework for taking a pertinent history from a patient who wishes to fast.
4. Generate ideas for altering medical advising to accommodate lifestyle changes during Ramadan.

Citation:
Khan S, Siraj Z, Ata A, Azam N. Clinical considerations while providing care for patients during Ramadan: a framework for health care professionals. *MedEdPORTAL.* 2026;22:11614.
https://doi.org/10.15766/mep_2374-8265.11614

Introduction

Ramadan, the holiest month of the Islamic calendar, commemorates when the Holy Quran was revealed to the Prophet Muhammed. Fasting during Ramadan, 1 of the 5 pillars of Islam, is among the most important religious duties for Muslims. During this time, Muslims try to attain *taqwa*, or God consciousness, in everything they do and strive to keep spirituality at the forefront of their mind and actions throughout each day. For example, they may strive to refrain from sins such as backbiting, lying, or using profanity. During Ramadan, patients must abstain from all food, water, and medications from sunrise to sunset; they must also abstain from smoking during the month. It also leads to changing sleep hours to complete all obligatory and supererogatory prayers and to observe Suhoor, the predawn meal consumed before fasting during the month. The time of year is dependent on the lunar calendar and

Copyright © 2026 Khan et al. This is an open-access publication distributed under the terms of the Creative Commons Attribution license.

sighting of the new moon but occurs about 10 days earlier each year.

In Islam, Muslims who have chronic conditions, are pregnant, or are breastfeeding may be exempt from fasting if it would cause them undue harm. These decisions are ideally made in consultation with a health care provider, yet many Muslims do not engage their clinicians in such discussions. Instead, patients often rely on religious leaders, family, or community members for guidance, and some make independent changes to their care altogether. Interviews with Muslims who have chronic diseases show that many continue fasting despite risks, citing spiritual fulfillment, fear of guilt, or societal pressures.[1] Additionally, numerous studies have reported that Muslim patients will alter their medication regimen or stop taking medications altogether during Ramadan.[1-3] A retrospective review of electronic health records and billing statements in Massachusetts found that hospitalizations, primary care visits, and emergency room visits were higher for Muslims than non-Muslims during Ramadan, largely related to exacerbations of chronic disease.[2]

Barriers to patient-provider communication further complicate this issue. Upon questioning Muslim pregnant patients in the United States, patients report avoiding consulting their clinician about fasting "due to past negative provider experiences, fear of disrespectful treatment, or being given a blanket prohibition on fasting without well-informed explanations."[4] Conversely, patients are more likely to follow the advice of physicians who are knowledgeable and respectful of their beliefs.[2] Still, many providers are unfamiliar with the details of religious practice during Ramadan and rarely counsel patients on managing lifestyle changes during this time. In a study conducted in Nova Scotia, more than half of physicians surveyed did not know that Muslims could not drink water or take medications during their fast.[5] Similarly, a study in New York City found that most physicians treating diabetic patients during Ramadan did not adjust patient medication regimens or blood sugar monitoring to accommodate their fast, discuss diabetes complications related to fasting, or even inquire about fasting with their patients.[6]

Evidence suggests that tailoring care to Ramadan practices can improve outcomes. For example, implementing evening home delivery of tuberculosis medications increased treatment completion among Muslim patients fasting for Ramadan to rates comparable with non-Muslims.[7] Empowering physicians with knowledge about Ramadan can allow them to be more in tune to the needs of their patients and better equipped to provide relevant medical advice to improve their health.

To address improving provider knowledge of Ramadan, we developed and implemented an educational workshop aimed at increasing comfort in managing patients who fast, and empowering clinicians to adjust medical advice in alignment with patients' religious obligations. In the long term, this approach seeks to enhance communication, foster trust, and improve health outcomes for Muslim patients during Ramadan. Previous studies published by *MedEdPORTAL* focus on the intersection of health care and Islam in general; however, this work looks specifically at the effects of fasting for Ramadan and physiologic changes occurring during fasting for patients with chronic conditions.[8,9]

Methods

We offered a 1-hour workshop to health care professionals associated with Albany Medical College and Albany Medical Center and its partner sites during Ramadan in March 2025. We offered workshops to 4 distinct groups: (1) family medicine residents and rotating medical students, (2) second-year medical students transitioning to clinical rotations, (3) all employees of a local community health center outside of the academic medical center, and (4) internal medicine residents. We scheduled these workshops during supplemental in-person didactic time and designed, led, and facilitated by medical student authors (SK and ZS). This project was deemed exempt by the Albany Medical College Institutional Review Board.

Workshop Content

Workshop development was informed by Transcultural Nursing Theory developed by Madeleine Leininger, also known as the Sunrise Model framework. The Sunrise Model emphasizes that factors such as religion, kinship and family structure, cultural values, prior health care experiences, and broader social context shape how patients interact with health care systems.[10] This aligns with the concept of "culturally congruent care," first described by Leininger in the mid-20th century, which emphasizes that health care should be delivered within the context of a patient's belief system that contributes to their broader worldview.[11] Embedded within the culturally congruent care approach is the idea that clinicians must recognize the limits of their own cultural assumptions to respectfully engage patients and adapt care through shared decision-making.

Prior work has applied Leininger's framework across diverse health care settings and cultural contexts, including the care of Muslim patients.[4,12] Literature regarding Ramadan fasting further highlights the importance of culturally responsive health care communication and treatment planning surrounding fasting

Copyright © 2026 Khan et al. This is an open-access publication distributed under the terms of the Creative Commons Attribution license.

during illness. Moreover, there is an emphasis on the need for culturally humble environments in which Muslim patients feel heard and supported, the absence of which serves as a barrier to seeking medical guidance.[2]

The session was guided by a detailed facilitator guide, which offers instructions on workshop pacing and content with direct quotations on how to deliver the material (Appendix A), and a PowerPoint presentation, which acts as a visual guide for the workshop and contains QR codes to key resources for participants (Appendix B). It began with an overview of Ramadan, including its religious significance, fasting requirements and exemptions, and typical prayer/fasting schedules. Discussion then addressed the clinical implications of fasting, particularly for patients with chronic conditions who may not consult providers when altering their management. Pregnancy, chronic kidney disease, and diabetes were highlighted as exemplar conditions requiring modified care plans during Ramadan. Recommendations for patients' diets were provided.[13] Participants were presented with examples of patient scenarios and tasked with generating ideas for altering advice to best accommodate the patient's needs while fasting, first as a large group, then in small groups of 3-4 self-assembled individuals. Although the facilitators of these workshops are Muslim, facilitators do not need to be Muslim if they have sufficient understanding of the workshop content and feel comfortable offering advice to participants. Non-Muslim facilitators should thoroughly review the facilitator guide and can consult spiritual leaders online or at a local mosque for greater understanding of the religious significance or lifestyle changes during Ramadan.

To equip participants for patient-centered discussions, we used a structured framework for history-taking and shared decision-making. The framework included exploration of medical history, intended lifestyle changes, values regarding fasting, fasting duration and climate, prior fasting experiences, medication regimens, alarming symptoms, and available resources. Participants then applied this framework to case studies (chronic kidney disease, hypothyroidism, and type 2 diabetes), generating management strategies that were subsequently reviewed in group discussion. Previous literature details frameworks to determine the risk associated with patient fasting, but this workshop builds on that literature to help clinicians understand how to utilize this preexisting framework to get a greater appreciation for the changes in their patients' lives, apply their medical knowledge to advise their patients, and make shared decisions about what is best for the patient.[14]

Pre- and postworkshop surveys (Appendix C) were administered using Qualtrics XM. The presurvey captured participant demographics, baseline knowledge of Ramadan, prior experience caring for fasting patients, and comfort in providing care and was administered prior to reviewing any content or learning objectives. The postsurvey repeated these measures to assess changes in knowledge and confidence and was administered after addressing any audience questions. Surveys were accessed via QR codes displayed on presentation slides and completed on mobile devices. Any survey responses that were missing their preworkshop or postworkshop pair were not used in data analysis.

Data Analysis

Knowledge was assessed using true/false questions on basic Ramadan and fasting practices. Comfort was measured through self-reported ratings on a 5-point Likert scale addressing 3 domains: discussing fasting practices with patients, adjusting medication regimens, and understanding how fasting impacts health. Pre- and postworkshop responses were compared using McNemar's chi-square test. For knowledge-based items, the number of correct versus incorrect responses was compared between time points. For comfort-based items, responses of *disagree*, *somewhat disagree*, or *neutral* were grouped and compared against responses of *somewhat agree* or *agree* to evaluate changes in comfort levels. Significant differences between the pre- and postworkshop surveys represent level 2 on Kirkpatrick's model of training evaluation, showing that they have sufficiently learned the concepts taught in the workshop. Health care professional application of concepts taught in the workshops and subsequent changes in patient outcomes were not measured in this study.

Results

A total of 76 participants were present, and a total of 54 matched pre- and postworkshop survey responses were collected across 4 sessions, leading to a 71% response rate. Participants included medical students, residents, attending physicians, nurses, and other health care professionals within and throughout the academic health care system.

Demographics

Participant characteristics are summarized in Table 1. Most respondents identified as White/Caucasian (59.3%), followed by Asian/Asian American (14.8%), South Asian/South Asian American (9.3%), Black/African American (3.7%), Arab/Arab American (3.7%), Hispanic/Latino (1.9%), and other (7.4%). Regarding gender, 66.7% identified as female, 29.6% identified as

Copyright © 2026 Khan et al. This is an open-access publication distributed under the terms of the Creative Commons Attribution license.

Table 1. Demographic Information of Workshop Participants ($N = 54$)

Demographic	No. (%)
Race	
Arab/Arab American	2 (3.7)
Asian/Asian American	8 (14.8)
Black/African American	2 (3.7)
Hispanic/Latino	1 (1.9)
Indigenous American/Alaskan Native	0 (0)
South Asian/South Asian American	5 (9.3)
White/Caucasian	32 (59.3)
Other	4 (7.4)
Gender	
Male	16 (29.6)
Female	36 (66.7)
Nonbinary/third gender	0 (0)
Prefer not to say	2 (3.7)
Level of training	
Attending	1 (1.9)
Fellow	0 (0)
Resident	18 (33.3)
Fourth-year medical student	0 (0)
Third-year medical student	4 (7.4)
Second-year medical student	13 (24.1)
First-year medical student	0
Other health care professional	18 (33.3)
Religion	
Agnostic	2 (3.7)
Atheist	5 (9.3)
Christianity	22 (40.7)
Islam	5 (9.3)
Hinduism	7 (12.9)
Judaism	2 (3.7)
Other	6 (11.1)
None	5 (9.3)
Religiosity	
Very religious	4 (7.4)
Moderately religious	9 (16.7)
Somewhat religious	18 (33.3)
Not religious at all	17 (31.5)
Other	3 (5.6)
Prefer not to say	3 (5.6)
Previously participated in a fast for Ramadan	
Yes	15 (27.8)
No	39 (72.2)

male, and 3.7% preferred not to answer. Participants' professional roles included attending physicians (1.9%), residents (33.3%), medical students (31.5%), and other health care professionals (33.3%).

In terms of religion, 40.7% identified as Christian, 12.9% as Hindu, 9.3% as Muslim, 9.3% as atheist, 3.7% as agnostic, 3.7% as Jewish, and 11.1% as other religions, and 9.3% reported no religious affiliation. Religiosity varied, with 7.4% identifying as very religious, 16.7% as moderately religious, 33.3% as somewhat religious, 31.5% as not religious, and 5.6% as other, and 5.6% preferring not to answer. A minority of participants (27.8%) reported ever observing a fast for Ramadan independent of their previously stated religious affiliation.

Overall, the demographic distribution indicated that the audience primarily represented majority identities.

Knowledge Outcomes

Participant knowledge of Ramadan practices was assessed using true/false questions. Participants generally displayed a baseline understanding that fasting involves abstaining from food and drink from sunrise to sunset ($P > .05$) and that exemptions exist for individuals with chronic conditions ($P > .05$); these did not significantly change following the workshop. However, significant improvements were observed in other domains. Participants demonstrated increased understanding that water is prohibited during fasting ($P < .05$), that oral medications and IV fluids invalidate the fast ($P < .05$), and that Ramadan does not occur at the same time each year ($P < .05$). Overall, the workshop produced statistically significant gains in knowledge of Ramadan-related religious practices (Table 2).

Comfort Outcomes

Participants also demonstrated significant increases in comfort providing care for patients fasting during Ramadan when comparing pre- and postworkshop survey responses of participants. To evaluate changes in comfort providing care to fasting patients, Likert-scale responses were collapsed into 2 categories: *strongly disagree*, *disagree*, and *neutral* were grouped together, and *agree* and *strongly agree* were grouped together. Postworkshop, respondents reported greater comfort in directly or indirectly asking patients about fasting intentions ($P < .05$), providing care to fasting patients ($P < .05$), and caring for fasting patients with underlying conditions ($P < .0001$). They also reported increased comfort with obtaining relevant medical and social histories ($P < .05$), adjusting medication regimens for fasting patients ($P < .05$), and advocating on behalf of fasting patients to other health care providers ($P < .05$). These improvements are highly meaningful, as they can lead to direct changes in patient care and more open conversations between patients and providers. Additionally, participants showed improved ability to identify resources to guide clinical decision-making for fasting patients[15-18] ($P < .05$) and greater understanding of how religious practices affect health ($P < .05$) in the postworkshop responses compared to the preworkshop responses. No significant changes were observed in understanding that religious beliefs play an important role in patient care ($P > .05$) or that fasting can influence medical advice ($P > .05$), likely due to high baseline awareness by practicing in a geographic area with a high Muslim population. Overall, the workshop was associated with significant improvements in provider comfort and confidence in managing fasting patients (Table 3).

Copyright © 2026 Khan et al. This is an open-access publication distributed under the terms of the Creative Commons Attribution license.

Table 2. Workshop Participants' Self-Reported Knowledge of Religious Practices for Ramadan (*N* = 54)

Item Assessed Via True/False Question	Preworkshop Response (%)	Postworkshop Response (%)	*P*
Knows that people fasting for Ramadan must abstain from eating or drinking from sunrise to sunset	96.0	100.0	.5
Knows that people fasting for Ramadan must abstain from drinking water from sunrise to sunset	67.0	100.0	<.0001
Knows that people fasting for Ramadan must abstain from taking oral medications or IV fluids from sunrise to sunset	38.9	98.2	<.001
Knows that some practicing Muslims may be exempt from fasting during Ramadan due to chronic conditions	92.6	92.6	<.99
Knows that Ramadan takes places at a different time each year	74.0	94.4	.0034

Discussion

This workshop was effective in increasing health care providers' knowledge of Ramadan practices and in improving comfort with tailoring medical advice for fasting patients with chronic disease. Notably, participants reported greater confidence in adjusting medication regimens and in identifying resources to guide clinical decision-making. While behavior changes were not measured in this study, increased knowledge of these concepts can empower health care professionals to have more open conversations with patients by proactively asking patients about fasting plans and changing clinical advice accordingly.

As cultural humility gains importance in medical education, it is increasingly recognized that providers cannot be experts in every cultural or religious tradition. Instead, they must develop skills to elicit individual patients' beliefs and practices to deliver care that is both realistic and respectful. Given that Islam is the second largest world religion, and the Muslim population continues to grow globally, providers are likely to encounter Muslim patients who wish to fast during Ramadan. Although many participants had a basic awareness of Ramadan, the workshop revealed gaps in recognizing when and how medical advice should be modified. This is especially important for primary care providers, who served as the main audience for this workshop, as they are often the first point of contact for patients with chronic disease and are best positioned to integrate social and religious considerations into care.

Leininger's Sunrise Model influenced the development of this workshop to help providers understand how to provide culturally congruent care. This model was developed to improve transcultural nursing care but can be adopted to assist all health care professionals in understanding how a patient's values, beliefs, and lifestyle affect their practices and attitudes toward health care.[19] This workshop focuses on exploring broader topics such as Islam and Ramadan as a whole and progresses toward topics focusing on health care by looking at specific conditions and patient cases. This is meant to help professionals support patient values and adapt their daily habits for a more salutogenic lifestyle.

The use of case-based learning allowed participants to explore how fasting can affect outcomes in conditions such as diabetes, chronic kidney disease, and pregnancy. This approach helped participants generate practical strategies while also fostering empathy for patients who prioritize fasting as a religious obligation. By encouraging providers to engage in collaborative decision-making, this curriculum supports stronger patient–

Table 3. Participants' Self-Assessed Comfort and Understanding of Patients Fasting for Ramadan (*N* = 54)

Item[a]	Preworkshop Agreement[b] (%)	Postworkshop Agreement[b] (%)	*P*
Comfortable asking patients if they are fasting for Ramadan	70.4	94.4	.0002
Comfortable providing care to patients fasting for Ramadan	66.7	94.4	.0001
Comfortable providing care to patients with underlying conditions who are fasting for Ramadan	59.3	90.7	<.0001
Comfortable obtaining a relevant social and medical history from patients who are fasting for Ramadan	72.2	94.4	.042
Comfortable adjusting medications/medication use practices for patients who are fasting for Ramadan	40.7	75.9	<.0001
Comfortable advocating on behalf of a patient who is fasting for Ramadan to another health care provider who is unaware of the patient's current practices	79.6	96.3	.0039
Understands that a patient's religious beliefs and values is an important factor in providing care and meeting their health care needs	92.6	98.1	.375
Understands that fasting for Ramadan may impact the way that I advise patients to manage their health	81.5	96.3	.215
Able to find resources that will provide information on how to effectively manage patients fasting for Ramadan	50.0	96.3	<.0001
Understands how religious practices for Ramadan can impact patient health	70.4	98.2	.0003

[a]Rated based on a 5-point Likert scale (1 = *strongly disagree*, 2 = *disagree*, 3 = *neutral*, 4 = *agree*, 5 = *strongly agree*).
[b]The percentage combines *agree* and *strongly agree* responses.

Copyright © 2026 Khan et al. This is an open-access publication distributed under the terms of the Creative Commons Attribution license.

provider trust and may contribute to improved chronic disease control during Ramadan.

An important consideration raised during the workshop was the diversity of religious observance within Islam. While general principles and common practices can be taught, each patient interprets and practices their faith differently. Participants expressed some uncertainty in applying uniform guidance to complex or urgent scenarios. To address this, the workshop emphasized individualized discussions with patients regarding their practices and expectations. Future iterations of this curriculum should incorporate structured engagement with local religious leaders and community organizations. Such partnerships could strengthen provider knowledge, improve accessibility of tailored care, and allow proactive planning in the weeks leading up to Ramadan.

This study has several limitations. First, participation was limited to 4 departments based on those who responded to email invitations, resulting in a modest sample size. Technical issues, such as lack of access to technology or inability to scan QR codes, prevented some participants from completing online surveys, further reducing statistical power. This problem can be addressed in the future by offering paper copies of the surveys and handouts in addition to the online versions to accommodate those without ready access to devices connected to the internet.

Second, representation across provider groups was uneven. Although residents and medical students were well represented, few attendings or fellows participated due to competing clinical and teaching responsibilities. Moreover, participation was limited to certain medical student cohorts due to scheduling and lack of availability of both first- and fourth-year medical students. As a result, the majority of participants were trainees with variable prior experience in patient care. Future studies should expand the inclusion of attendings and fellows to evaluate whether greater clinical experience influences knowledge acquisition and confidence. This may be accomplished by including incentives for workshop participation, such as continuing education credits.

Third, although fasting affects management across multiple specialties, participants were primarily drawn from primary care disciplines and medical education settings. Future work should explore how specialty providers can complement primary care in counseling fasting patients and develop coordinated, interdisciplinary strategies. Expert resources on specific conditions were shared with participants, but time constraints limited the review of these materials. Increased workshop length could allow for greater interdisciplinary discussion and literature review.

Finally, the workshops were conducted during Ramadan to maximize relevance, but ideally such interventions should be delivered in the months preceding Ramadan to allow time for shared decision-making and care planning. Future research should evaluate real-world outcomes by assessing provider–patient conversations, patient experiences, and clinical outcomes following implementation of this curriculum. This would also help mitigate any bias in survey responses from individuals over- or underestimating their confidence or abilities gained from the workshop by allowing for analysis of the actions taken as a result of what was learned.

Appendices

A. Facilitator Guide.docx

B. Ramadan and the Fasting Patient Module.pptx

C. Preworkshop and Postworkshop Survey.docx

All appendices are peer reviewed as integral parts of the Original Publication.

Summer Khan: Fourth-Year Medical Student, Albany Medical College; ORCID: https://orcid.org/0009-0001-7049-0522

Zaina Siraj, MD: Resident Physician, Department of Internal Medicine, Albany Medical Center

Ashar Ata, MPH, PhD: Statistician, Department of Surgery, Albany Medical Center

Nyla Azam, MD: Assistant Professor, Department of Anesthesiology, Albany Medical Center

Disclosures
None to report.

Funding/Support
None to report.

Prior Presentations
Khan S, Siraj Z, Ata A, Azam N. Advancing Ramadan-specific patient care: evidence from a health care professional training session. Poster presented at: Albany Medical College Medical Student Investigation Day; October 1, 2025; Albany, NY.

Khan S, Siraj Z, Ata A, Azam N. Advancing Ramadan-specific patient care: evidence from a health care professional training session. Poster presented at: AMMSA 2025 National Conference; October 31–November 2, 2025; Richardson, TX.

Copyright © 2026 Khan et al. This is an open-access publication distributed under the terms of the Creative Commons Attribution license.

Ethical Approval

The Albany Medical College Institutional Review Board reviewed this project.

References

1. Alshehri AM, Barner JC, Wong SL, Ibrahim KR, Qureshi S. Perceptions among Muslims regarding fasting, medication use and provider engagement during Ramadan in the United States. *Int J Health Plann Manage*. 2021;36(3):945-957. https://doi.org/10.1002/hpm.3147
2. Zagloul M, Mohammed B, Abufares N, et al. Review of Muslim patient needs and its implications on healthcare delivery. *J Prim Care Community Health*. 2024;15:21501319241228740. https://doi.org/10.1177/21501319241228740
3. Hamodat H, Syed S, Ali M, et al. Primary care physicians' knowledge, perceptions, and comfort level in managing patients fasting in Ramadan. *J Prim Care Community Health*. 2020;11: 2150132720933796. https://doi.org/10.1177/2150132720933796
4. Wehbe-Alamah H. Bridging generic and professional care practices for Muslim patients through use of Leininger's culture care modes. *Contemp Nurse*. 2008;28(1-2):83-97. https://doi.org/10.5172/conu.673.28.1-2.83
5. Hillier KAW, Longworth ZL, Vatanparast H. Healthcare professionals knowledge, attitude, practices, and perspectives providing care to Muslims in Western countries who fast during Ramadan: a scoping review. *Appl Physiol Nutr Metab*. 2024;49(4):415-427. https://doi.org/10.1139/apnm-2023-0462
6. Ali M, Adams A, Hossain MA, Sutin D, Han BH. Primary care providers' knowledge and practices of diabetes management during Ramadan. *J Prim Care Community Health*. 2016;7(1): 33-37. https://doi.org/10.1177/2150131915601359
7. Stockbridge EL, Kabani FA, Gallups JS, Miller TL. Ramadan and culturally competent care: strengthening tuberculosis protections for recently resettled Muslim refugees. *J Public Health Manag Pract*. 2020;26(5):E13-E16. https://doi.org/10.1097/PHH.0000000000000893
8. Sarsour NY, Hammoud MM. Integration of Arab and Muslim health education into a medical school curriculum. *MedEdPORTAL*. 2021;17:11188. https://doi.org/10.15766/mep_2374-8265.11188
9. Stoller L, Blanchard E, Fowler M. Trigger topics: where religion & health care intersect. *MedEdPORTAL*. 2015;11:10007. https://doi.org/10.15766/mep_2374-8265.10007
10. McFarland MR, Wehbe-Alamah HB. Leininger's theory of culture care diversity and universality: an overview with a historical retrospective and a view toward the future. *J Transcult Nurs*. 2019;30(6):540-557. https://doi.org/10.1177/1043659619867134
11. Gonzalo A. Madeleine Leininger: transcultural nursing theory. *Nurseslabs*. Updated April 30, 2024. Accessed May 12, 2026. https://nurseslabs.com/madeleine-leininger-transcultural-nursing-theory/
12. Luna L. Care and cultural context of Lebanese Muslim immigrants: using Leininger's theory. *J Transcult Nurs*. 1994; 5(2):12-20. https://doi.org/10.1177/104365969400500203
13. Khalife T, Pettit JM, Weiss BD. Caring for Muslim patients who fast during Ramadan. *Am Fam Physician*. 2015;91(9):641-642.
14. Mahmood A, Dar S, Dabhad A, Aksi B, Chowdhury TA. Advising patients with existing conditions about fasting during Ramadan. *BMJ*. 2022;376:e063613. https://doi.org/10.1136/bmj-2020-063613
15. Waqar S, Ghouri N, Awaad R. Chronic disease management during Ramadan. *Am Fam Physician*. 2023;107(2):125-126.
16. Shahawy S, Al Kassab L, Rattani A. Ramadan fasting and pregnancy: an evidence-based guide for the obstetrician. *Am J Obstet Gynecol*. 2023;228(6):689-695. https://doi.org/10.1016/j.ajog.2023.03.023
17. British Islamic Medical Association. Ramadan compendium. *BIMA*. Updated February 2023. Accessed May 11, 2026. https://britishima.org/advice/ramadan-compendium/
18. Hassanein M, Afandi B, Yakoob Ahmedani M, et al. Diabetes and Ramadan: practical guidelines 2021. *Diabetes Res Clin Pract*. 2022;185:109185. https://doi.org/10.1016/j.diabres.2021.109185
19. Farokhzadian J, Nematollahi M, Dehghan Nayeri N, Faramarzpour M. Using a model to design, implement, and evaluate a training program for improving cultural competence among undergraduate nursing students: a mixed methods study. *BMC Nursing*. 2022;21(1)85. https://doi.org/10.1186/s12912-022-00849-7

Received: November 13, 2025
Accepted: May 14, 2026
Published: June 25, 2026

Copyright © 2026 Khan et al. This is an open-access publication distributed under the terms of the Creative Commons Attribution license.
